# Novel Genetic Locus Influencing Retinal Venular Tortuosity Is Also Associated With Risk of Coronary Artery Disease

**DOI:** 10.1161/ATVBAHA.119.312552

**Published:** 2019-10-10

**Authors:** Abirami Veluchamy, Lucia Ballerini, Veronique Vitart, Katharina E. Schraut, Mirna Kirin, Harry Campbell, Peter K. Joshi, Devanjali Relan, Sarah Harris, Ellie Brown, Suraj S. Vaidya, Baljean Dhillon, Kaixin Zhou, Ewan R. Pearson, Caroline Hayward, Ozren Polasek, Ian J. Deary, Thomas MacGillivray, James F. Wilson, Emanuele Trucco, Colin N.A. Palmer, Alexander S.F. Doney

**Affiliations:** 1 From the Division of Population Health and Genomics (A.V., E.R.P., C.N.A.P., A.S.F.D.), University of Dundee, United Kingdom; 2 Ninewells Hospital and Medical School and VAMPIRE project, Computer Vision and Image Processing Group, School of Science and Engineering (Computing) (L.B., E.T.), University of Dundee, United Kingdom; 3 MRC Human Genetics Unit (V.V., C.H., J.F.W.), MRC Institute of Genetics and Molecular Medicine, University of Edinburgh, Western General Hospital, United Kingdom; 4 Medical Genetics Section, Centre for Genomic and Experimental Medicine (S.H.), MRC Institute of Genetics and Molecular Medicine, University of Edinburgh, Western General Hospital, United Kingdom; 5 Centre for Global Health Research, Usher Institute of Population Health Sciences and Informatics, University of Edinburgh, Scotland, United Kingdom (K.E.S., M.K., H.C., P.K.J., J.F.W.); 6 Centre for Cardiovascular Science (K.E.S.), Queen’s Medical Research Institute, University of Edinburgh, Royal Infirmary of Edinburgh, Scotland, United Kingdom; 7 Clinical Research Imaging Centre (E.B., S.S.V.), Queen’s Medical Research Institute, University of Edinburgh, Royal Infirmary of Edinburgh, Scotland, United Kingdom; 8 VAMPIRE project, Centre for Clinical Brain Sciences, Chancellor’s Building, Royal Infirmary of Edinburgh, Scotland, United Kingdom (L.B., D.R., B.D., T.M.); 9 Department of Psychology (I.J.D.), University of Edinburgh, United Kingdom; 10 Centre for Cognitive Ageing and Cognitive Epidemiology (S.H., I.J.D.), University of Edinburgh, United Kingdom; 11 Department of Psychology (S.H.), University of Edinburgh, United Kingdom; 12 Department of Public Health, University of Split, School of Medicine, Croatia (M.K., O.P.); 13 Renji Hospital, University of Chinese Academy of Sciences, Chongqing, China (K.Z.); 14 Department of Computer Science, BML Munjal University, Gurgaon, Haryana, India (D.R.).

**Keywords:** atrial fibrillation, biomarkers, cardiovascular diseases, genome-wide association study, heart rate, retina

## Abstract

The retina may provide readily accessible imaging biomarkers of global cardiovascular health. Increasing evidence suggests variation in retinal vascular traits is highly heritable. This study aimed to identify the genetic determinants of retinal vascular traits.

**Approach and Results:**

We conducted a meta-analysis of genome-wide association studies for quantitative retinal vascular traits derived using semi-automatic image analysis of digital retinal photographs from the GoDARTS (Genetics of Diabetes Audit and Research in Tayside; N=1736) and ORCADES (Orkney Complex Disease Study; N=1358) cohorts. We identified a novel genome-wide significant locus at 19q13 (*ACTN4/CAPN12*) for retinal venular tortuosity (*TortV*), and one at 13q34 (*COL4A2*) for retinal arteriolar tortuosity (*TortA*); these 2 loci were subsequently confirmed in 3 independent cohorts (N_total_=1413). In the combined analysis of discovery and replication cohorts, the lead single-nucleotide polymorphism in *ACTN4*/*CAPN12* was rs1808382 (β_s.d._=-0.109; SE=0.015; *P*=2.39×10-^13^) and in *COL4A2* was rs7991229 (β_s.d._=0.103; SE=0.015; *P*=4.66×10-^12^). Notably, the *ACTN4*/*CAPN12* locus associated with *TortV* is also associated with coronary artery disease, heart rate, and atrial fibrillation.

**Conclusions:**

Genetic determinants of retinal vascular tortuosity are also linked to cardiovascular health. These findings provide a molecular pathophysiological foundation for the use of retinal vascular traits as biomarkers for cardiovascular diseases.

## Abstract 2

Supplemental Digital Content is available in the text.

**Highlights**

- Emerging evidence indicates that retinal tortuosity traits are associated with vascular health and highly heritable. However, the genetic architecture of retinal vascular tortuosity has not been investigated.
- By using a meta-analysis of genome-wide association studies, we found a novel association at 19q13 (*ACTN4/CAPN12*) for retinal venular tortuosity (*TortV*), and one at 13q34 (*COL4A2*) for retinal arteriolar tortuosity (*TortA*) at discovery stage and validated in 3 independent cohorts.
- In-silico look-ups indicate that the significant associations between lead single-nucleotide polymorphisms at 19q13 and coronary artery disease, cardiovascular vascular risk factors, atrial fibrillation, and heart rate. Colocalization eQTL study (expression quantitative trait loci) found *CAPN12* as most likely to be a causal gene for *TortV* in heart and blood vessel tissues.
- Our findings highlight genetic impacts on *TortV*, and their association with cardiovascular disease and may provide a molecular pathophysiological foundation for the use of retinal vascular traits as biomarkers for cardiovascular diseases.

Retinal vascular traits can be readily measured noninvasively from fundus images, and changes in these traits have been linked to a number of clinical conditions associated with vascular health, including cardiovascular disease,^1,2^ stroke,^3^ hypertension,^4^ and neurodegenerative disease.^5^ The association between retinal vascular calibers and cardiovascular disease has been reported in numerous studies, and structural variation in retinal vasculature could predict cardiovascular risk.^6–8^ More recently, deep learning applied to retinal images has been successfully used to predict cardiovascular risk factors and outcomes.^9^ Whereas this potentially powerful approach indicated that vascular regions of the retina appeared important, it cannot provide a molecular pathoetiological basis for the link between retinal vasculature and cardiovascular disease.

Population-based studies have demonstrated a significant genetic component to variation in retinal blood vessel width.^10^ Evidence suggests that retinal vascular tortuosity, a potentially important vascular parameter, is also associated with a range of cardiovascular risk factors.^11,12^ Heritability estimates for retinal arterial tortuosity range from 50% to 82% and 21% for retinal venular tortuosity,^10,13^ indicating a substantial genetic contribution to the variation in these parameters. Recent genome-wide association studies (GWAS) found a number of loci for the more widely investigated retinal traits, central retinal vein equivalent (*CRVE*)^14–16^ and central retinal arteriolar equivalent (*CRAE*)^14–16^ as well as retinal image-derived optic disk morphology parameters.^17^

To our knowledge, no studies have performed a genome-wide scan on retinal vascular tortuosity traits. Understanding the molecular genetic architecture of retinal tortuosity traits would provide a molecular pathophysiological basis linking retinal microvascular features with systemic vascular pathology. We, therefore, performed a sufficiently powered GWAS to identify genetic variants influencing the retinal vascular tortuosity traits; arteriolar tortuosity (*TortA*), maximum *TortA* (*TortAmax*), venular tortuosity (*TortV*), and maximum *TortV* (*TortVmax*). We also examined other previously investigated retinal parameters, including *CRAE, CRVE*, arteriole-to-venule ratio (*AVR*), as well as the nonvascular optic disc radius (*ODradius*). We have investigated whether any of the significant variants associated with tortuosity traits are also associated with cardiovascular-related outcomes in the previously published GWAS results. There is ample evidence indicating that increased resting heart rate is linked to various cardiovascular events and associated with the increased risk of plaque rupture in coronary atherosclerosis patients.^18,19^ Therefore, we also checked whether there is any association between *TortV*-associated variants with heart rate in independent samples from United Kingdom Biobank.

## Subjects and Methods

Data analysis, methods, and all other supporting materials are available in the online-only Data Supplement document. The data that support the findings of this study are available to the researchers from the corresponding author upon request.

### Study Participants

Participants in the discovery phase of this study were obtained from 2 independent cohorts, the GoDARTS (Genetics of Diabetes Audit and Research in Tayside^20^) and the ORCADES (Orkney Complex Disease Study).^21^ Three independent cohorts of individuals of European ancestry were used at the replication stage, including the Lothian Birth Cohort 1936 (LBC1936),^22^ the Croatia-Korčula, and Croatia-Split study. Detailed descriptions of each cohort are presented in the online-only Data Supplement note.

### Retinal Vascular Parameter Measurement

Standard digital retinal photographs used for diabetes mellitus retinal screening were obtained from the clinical record of patients with type 2 diabetes mellitus in GoDARTS. A total of 1744 images (661 images from the GoDARTS data set 1 and 1083 images from GoDARTS data set 2) were selected for analysis after quality control (QC). Similarly, 1595 individual’s retinal images from ORCADES were used for this study after QC. The Vascular Assessment and Measurement Platform for Images of Retina 3.1, semi-automatic software, was used to measure retinal vascular traits in fundus images (Figure I in the online-only Data Supplement) from both GoDARTS and ORCADES. Standard protocols were followed to measure the retinal vessel parameters,^23^ including *CRAE*, *CRVE*, *AVR*, *ODradius*, *TortA*, *TortAmax*, *TortV*, and *TortVmax* (online-only Data Supplement). Tortuosity mean values were normalized by natural log transformation for association analysis (Figure II in the online-only Data Supplement). Smaller values indicate straighter vessels. After quality assessment and processing, a total of 644, 387, and 382 individuals’ retinal fundus images from the LBC1936, Croatia- Korčula, and Croatia-Split cohorts, respectively, were selected for the analysis. In these cohorts, retinal tortuosity traits were quantified using SIVA v3.1^24^ (Singapore I Vessels Assessment), semi-automated software, and normalized by natural log transformation for association analysis (online-only Data Supplement).

### Genotyping, QC, and Imputation

GoDARTS participants were genotyped using the Affymetrix 6.0 (n=927) and Illumina Human Omni Express (n=809) platforms. ORCADES samples were genotyped with either the Illumina HumanHap300 bead chip (n=890) or the Illumina Omni1M (n=304) or Illumina Omni Express bead chips (n=1073). Genotype data quality was assessed and imputed on the basis of 1000 Genome Projects reference panel. Imputed genotypes for 658, 1078, 1358 individuals from the GoDARTS data set 1, GoDARTS data set 2, and ORCADES cohorts, respectively, were used for the three independent GWAS analysis.

LBC1936 samples were genotyped using the Illumina Human 610Quad BeadChip. A total of 1398 participants from the 2 independent Croatian replication cohorts were available for the analysis, and subjects were genotyped on different genotyping platforms including Illumina CNV370v1 and CNV370-Quadv3 for Croatia-Korčula (n=378), and Illumina CNV370-Quadv3 and IlluminaOmniExpressExome-8v1_A for Croatia-Split (n=376). More details on QC, imputation, and processing can be found in the online-only Data Supplement.

### Statistical Analyses

We performed association analyses with each data set from GoDARTS separately for each of the 8 retinal traits using SNPTEST V2.5^25^ linear regression assuming an additive genetic model, adjusting for 3 ancestry principal components, age at eye examination, and gender. Association analysis in ORCADES was performed using linear mixed modeling to account for relatedness and assuming an additive genetic model, adjusting for 3 ancestry principal components, age and gender, using the mmscore function in ProbABEL.^26^ Then, we performed the meta-analysis using a fixed-effects model in GWAMA^27^ with the QC filtered GWAS summary results (imputation quality score >0.4 and minor allele frequency >0.03) from the GoDARTS and ORCADES. Manhattan plots, Quantile-Quantile plots, and forest plots were generated using in-built R scripts, and metafor—R package. Regional plots were generated using the Locus Zoom tool.^28^

Conditional analyses were performed in SNPTEST v2.5 using the genome-wide significant loci in the *COL4A2* region, conditioned on the lead single-nucleotide polymorphism (SNP) (rs56399312). In addition, this newly discovered locus was conditioned on previously reported genome-wide significant SNPs (rs4773144,^29^ rs11617955,^30^ and rs9515203^31^) associated with coronary artery disease (CAD). We performed an association test with an additive model adjusted for age, gender, and the first 3 principal components ancestry in the diabetes mellitus cohort (GoDARTS) using 759 samples without any cardiovascular events before the retinal screening date. We investigated the in-silico functional effects of the sentinel variants for each retinal vascular traits using various bioinformatics databases.^32–37^ Details can be found in the online-only Data Supplement.

The top 3 SNPs (*P*≤1.07×10^−07^) near *ACTN4*, *TMEM132*D, and *COL4A2* from the discovery stage for the tortuosity traits were taken forward for examination in 3 replication cohorts of European ancestry. In the LBC1936 cohort, association analyses were performed for arterial and venular tortuosity traits using linear regression model adjusting for age, sex, and 3 ancestry principal components, using mach2qtl. Similarly, in the Croatia—Split, and Korčula cohorts, association analyses were performed for each trait separately using linear mixed models implemented in the hglm R package, accounting for kinships derived using the gkin function of the GenABEL package.^38^

Summary association statistics for lead SNPs associated with *TortA* and *TortV* from the 2 discovery and 3 replication cohorts were combined and effect estimates from each cohort were presented in the forest plots using metafor—R package. Because of the variability in the β values and SE between the discovery and replication studies arising from different measurement algorithms, we standardized the effect estimates (using Cohen *d*) from each of the individual study cohorts. Cohen *d* is a scale-free interpretation which provides a standardized effect size measurement between the studies.^39^ In this study, Cohen *d* was calculated by the effect estimate divided by the SD.

### In-Silico Look-Ups of the Novel Variants for Clinical Outcomes

To investigate the association of the lead SNPs for *TortA*, and *TortV* with cardiovascular outcomes, we performed in-silico look-ups using summary association results from the Coronary Artery Disease Genome-wide Replication and meta-analysis plus C4D consortium,^29^ Global Lipid Genetics Consortium analysis^40^, and International consortium for blood pressure GWAS analysis.^41^ A recent study reported the association of *ACTN4* locus with heart rate.^42^ To examine whether the lead SNPs associated with *TortV* in *ACTN4* were also associated with heart rate, we checked the linkage disequilibrium (*r*^2^>0.8) between our SNPs and the index SNP (rs11083475) for heart rate in that study. Furthermore, we investigated the association of these SNPs with pulse rate in UK Biobank data.^43^ Additionally, we explored the relationship between the *TortV*-associated variants with atrial fibrillation (AF) using the summary statistics data from a recent large-scale meta-analysis of GWAS of AF.^44^ To identify the potential causal/target gene for *TortV*-associated variants, colocalization analysis was performed eCAVIAR.^45^ Details of these studies and online in-silico functional annotation resources have been described in the online-only Data Supplement on methods.

## Results

### Meta-Analysis of Discovery GWAS

**Figure 1.**
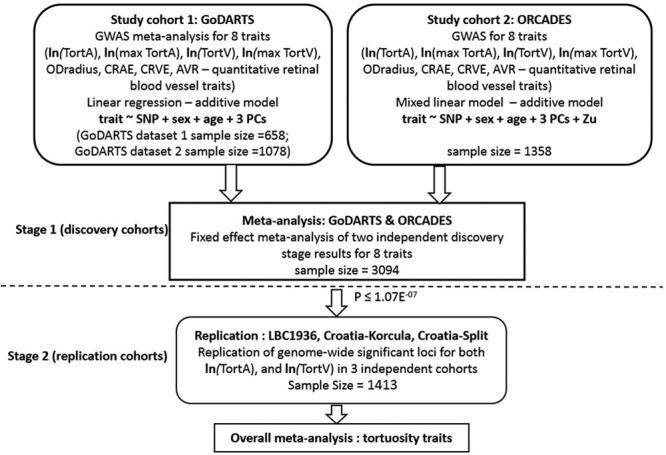
**Study Design.** Natural log-transformed data—*TortA, TortAmax, TortV, TortVmax*, u is the genetic value for each subject under a random-effects model, covariance amongst subjects assumed to be proportionate to the genomic relationship matrix. All Croatia indicates Croatia island of Korcula, Croatia-Split; *AVR*, arteriole-to-venule ratio; *CRAE*, Central Retinal Arteriolar Equivalent; *CRVE*, Central Retinal Venular Equivalent; GoDARTS, Genetics of Diabetes Audit and Research in Tayside; LBC1936, Lothian Birth Cohorts 1936; *ODradius*, Optic Disc Radius; ORCADES, Orkney Complex Disease Study; PC, principal components; *TortA*, retinal arteriolar tortuosity; *TortAmax*, maximum retinal arteriolar tortuosity; *TortV*, retinal venular tortuosity; and *TortVmax*, maximum retinal arteriolar tortuosity.

**Figure 2.**
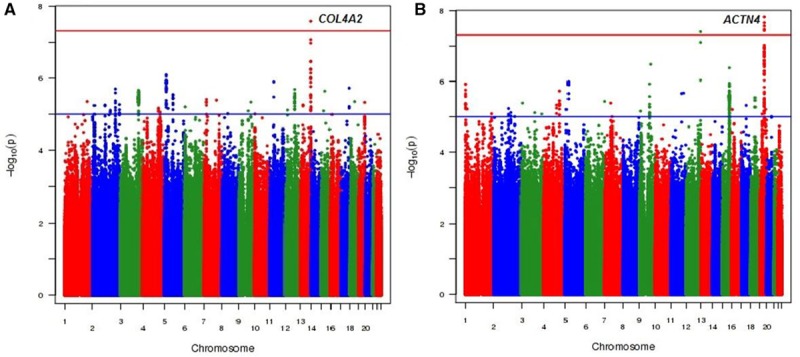
**Manhattan plots for meta-analysis of genome-wide association results from 2 independent discovery cohorts.** **A**, The results for the arteriolar tortuosity (*TortA*) and (**B**) represents the results for the venular tortuosity trait (*TortV*). The blue and red horizontal lines indicate the suggestive and genome-wide significance threshold (*P*<5×10^−8^), respectively.

**Figure 3.**
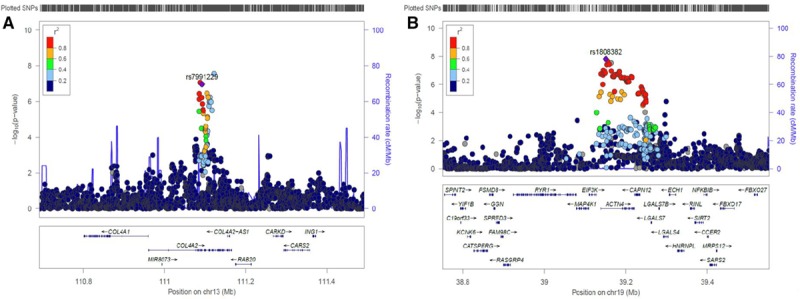
**Regional association and recombination plots of variants that reached *P* value <5×10^-7^ in the meta-analysis of the 2 discovery study cohorts (GoDARTS and ORCADES).** **A**, Lead SNP for *TortA*; **B**, lead SNP for *TortV*. Each plot was created using LocusZoom for the lead single-nucleotide polymorphism (SNP) in genomic region 400 kb in either side of the significant signal. Blue spikes represent the estimated recombination rates. Color scale (high to low *r*^2^) circles depicts the pairwise correlation (*r*^2^) between lead SNP and other SNPs in the loci. The lead SNP in that region is indicated by purple color solid diamond, and gene annotations in this region are shown in the **bottom**. GoDARTS indicates Genetics of Diabetes Audit and Research in Tayside; and ORCADES, Orkney Complex Disease Study.

The characteristics of the discovery study cohorts and overall study design can be found in Table I in the online-only Data Supplement and Figure 1. We combined the summary GWAS results from the GoDARTS and ORCADES cohorts for each trait using a fixed-effect meta-analysis, which overall showed no evidence of excessive amount of false-positive associations (genomic inflation factor of 0.99). Table II in the online-only Data Supplement presents the results from the meta-analysis and independent cohort GWAS analysis. Manhattan plots, Quantile-Quantile plots, and regional plots are shown in Figures 2 and 3 and Figures III through V in the online-only Data Supplement, respectively.

This analysis revealed one genome-wide significant (*P*<5×10^−8^) SNP, rs56399312, associated with *TortA* at 13q34, in *COL4A2* with moderate heterogeneity (*I*^2^=0.50; β=0.182, SE=0.032, *P*=2.70×10^−8^), and another SNP rs9515212 near *COL4A2* that was just below the threshold for genome-wide significance (β=0.151, SE=0.028, *P*=8.59×10^−8^). Conditional analysis on the lead SNP indicated that these are not independent signals (Table III in the online-only Data Supplement). Two genome-wide significant SNPs were associated with *TortV*, at 19q13 in *ACTN4* (lead SNP rs1808382; β=−0.123, SE=0.022, *P*=1.55×10^−8^; no heterogeneity, *I*^2^=0.00), and at 12q24.33 near *TMEM132D* (lead SNP rs73157566; β=−0.294, SE=0.054, *P*=4.07×10^−8^; low heterogeneity, *I*^2^=0.10); these associations at both these loci have not been reported previously with any retinal vascular parameters.

Moreover, we replicated 3 loci out of 8 previously reported loci for *CRVE*^14,16^ but did not replicate any of the previously reported SNPs associated with *CRAE*.^15^ Finally, we replicated a previously reported genome-wide significant locus for *ODradius* at 10q21.3 near *PBLD* (lead SNP rs61854835; β=−3.840, SE=0.575, *P*=4.06×10^−11^) and confirmed a number of other loci for this trait^17,46–48^ (Table IV in the online-only Data Supplement).

### Replication of Novel Associations With Vessel Tortuosity in Independent Cohorts

As candidates to carry forward for replication, we selected 3 lead SNPs from both loci (total 6 SNPs) (*ACTN4/CAPN12* and*TMEM132*D) for *TortV* and *COL4A2 for TortA* that reached significance *P*≤1.07×10^−07^ and had similar effect size and direction across the discovery cohorts. The characteristics of the replication cohorts are presented in Table V in the online-only Data Supplement.

Two *TortA*-associated SNPs, rs7991229, and rs9515212 in *COL4A2* reached nominal significance (*P*<0.05) in the LBC1936, Croatian cohorts. *P* values were significant after multiple testing in stage 2 and combined analyses (based on 2 independent tests). The lead SNP (rs56399312) from discovery stage did not reach significant *P* value in the LBC1936 but had similar effect size and direction. Two *TortV*-associated SNPs, rs1808382 and rs3786835 in *ACTN4/CAPN12* reached suggestive significance (*P*<1×10^−04^) in the combined analysis of replication cohorts whereas rs73157566 near *TMEM132D* did not replicate. Table contains the summary statistics from replication cohorts and meta-analysis of these cohorts.

**Table 1.**
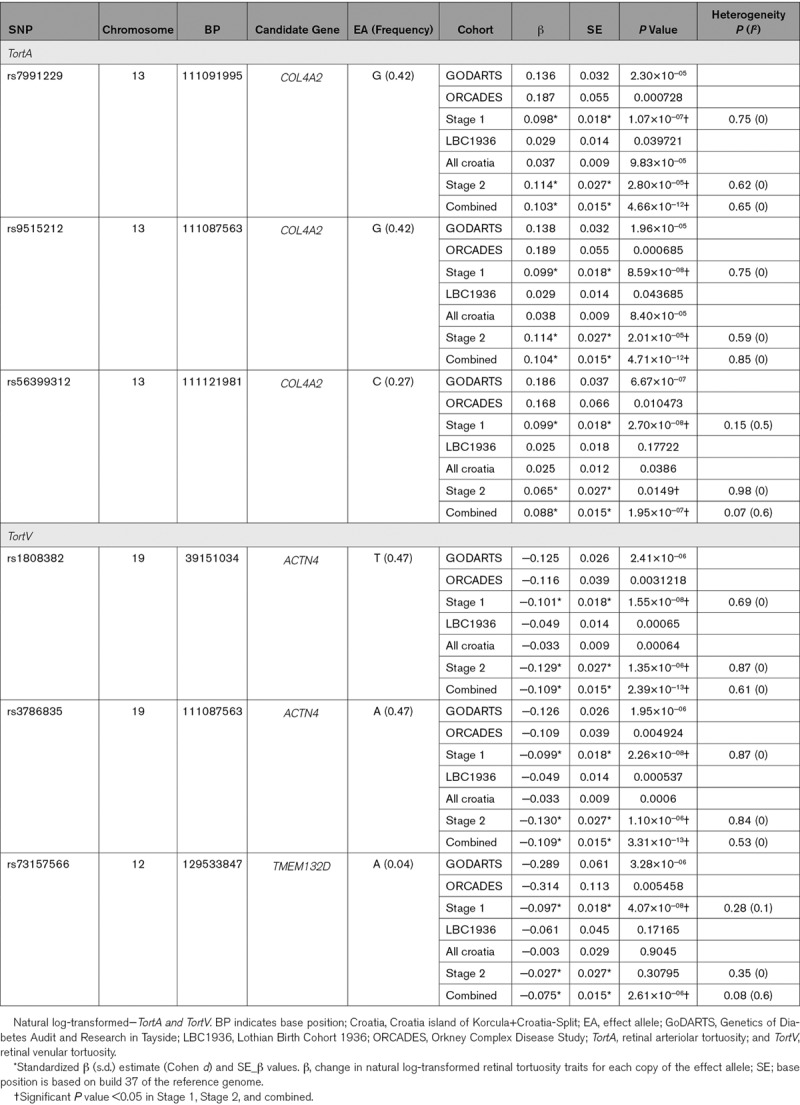
Results of Discovery, Replication, and Overall Meta-Analysis for Tortuosity Traits

### Meta-Analysis of Discovery and Replication Cohorts

**Figure 4.**
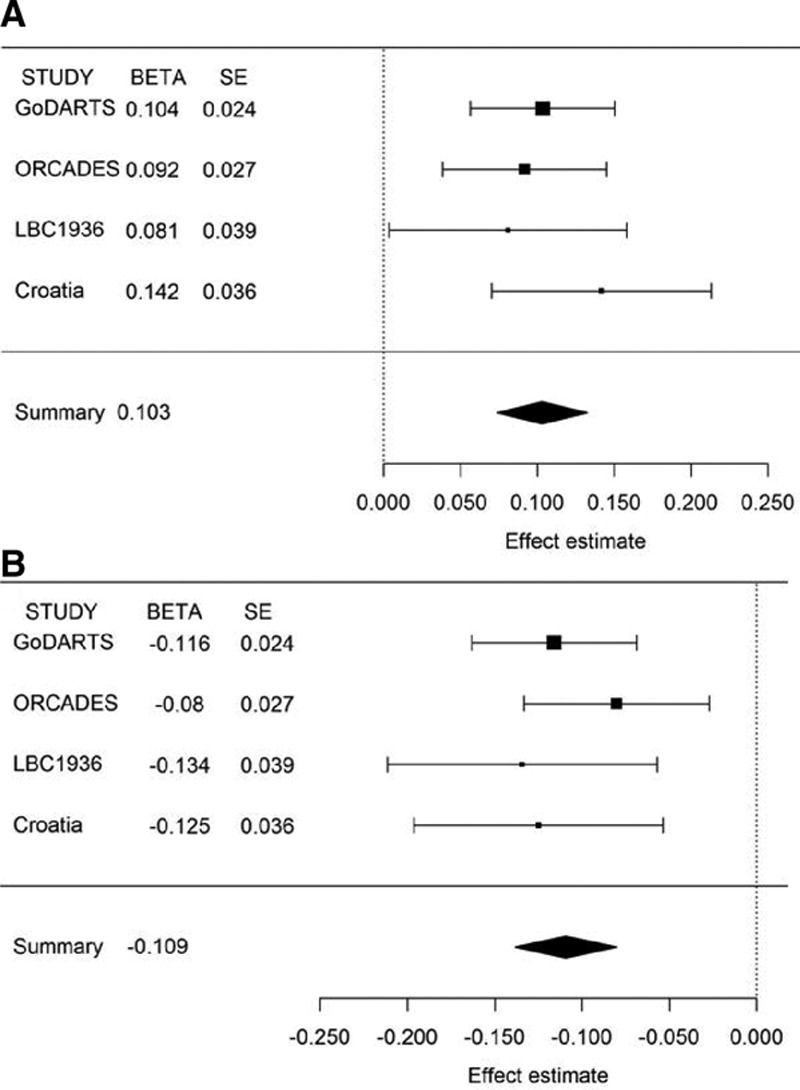
Forest plots for the genome-wide significant hits in overall meta-analysis. **A**, rs7991229 for *TortA*; **B**, rs1808382 for *TortV*. The plots represent standardized β (β_s__d_) and SE from GoDARTS (Genetics of Diabetes Audit and Research in Tayside), ORCADES (Orkney Complex Disease Study), LBC1936 (Lothian Birth Cohorts 1936), Croatia-Korcula Split, and meta-analysis study. Standardized β estimate: change in natural log-transformed retinal tortuosity traits for each copy of the effect allele.

In the overall meta-analysis, SNPs at 13q34, *COL4A2* (*TortA*) and 19q13, *ACTN4* (*TortV*) display genome-wide significant associations. Although *TortA*-associated SNPs, rs9515212 and rs7991229, were not genome-wide significant in the discovery meta-analysis, they reached genome-wide significance in the overall meta-analysis, and no heterogeneity (*I*^2^=0.00) was observed across different cohorts; *P*_overall_=4.66×10^−12^ and *P*_overall_=4.71×10^−12^, respectively. The lead SNP in *COL4A2* for *TortA* (rs56399312) at the discovery stage did not reach genome-wide significance in the overall meta-analysis (*P*_overall_=1.95×10^−07^), suggesting it is not likely to be the causal association at this locus that warrants further study. For *TortV* the lead SNPs, rs1808382 (*P*_overall_=2.39×10^−13^) and rs3786835 (*P*_overall_=3.31×10^−13^) near *ACTN4/CAPN12*, increased genome-wide significance and show no heterogeneity of effect sizes across studies (*I*^2^=0.00; Table). These SNPs are in tight linkage disequilibrium and, therefore, do not represent independent signals. The effect estimates from each of the individual study cohorts were standardized using Cohen *d*. Forest plots for lead SNPs in the combined analysis are shown in Figure 4.

### Functional Annotation: *TortA*-Associated Variants

*COL4A2* encodes collagen alpha-2(IV) (collagen type IV α 2), one of the 6 subunits of type IV collagens which are major structural components of basement membranes, forming a thin sheet of fibers under the endothelium controlling passage of vasoactive substances. These are conserved across species and C-terminal noncollagenous domains play a role in angiogenesis.^49^ Recent GWAS report that common variants around *COL4A2* and *COL4A1* (a paralogue immediately proximal to *COL4A2*, with which it shares a promoter and is co-expressed), are associated with coronary artery calcification,^50^ arterial stiffness,^51^ and CAD.^29–31,52^ Gene expression data from GeneAtlas,^53^ a human protein-coding transcriptome study validated the high expression of *COL4A2* in retinal microvessel endothelial cells (Figure VI in the online-only Data Supplement) whereas *COL4A1* is weakly expressed in retina indicating a specific role of *COL4A2* in the retinal vasculature. *TortA*-associated variants near *COL4A2* significantly alter transcription factor binding motifs and have putative effects on transcription as annotated by ENCODE (Table VI in the online-only Data Supplement). Additionally, expression data from the GTEx database^34^ confirmed that these significant SNPs are associated with the expression of *COL4A2* in heart left ventricle and artery aorta, shown in Table VII and Figure VII in the online-only Data Supplement, and these SNPs are in linkage disequilibrium (rs9515212 and rs7991229; *r*^2^=0.99, D′=1).

### Association of *TortA*-Associated Variants With Cardiovascular Risk Factors

Lead SNPs associated with *TortA* remained significant after conditioning on the previously reported cardiovascular risk variants in *COL4A2* (rs11617955,^29^ rs4773144,^30^ and rs9515203^31^; Figure VIII and Table VIII in the online-only Data Supplement). Conversely, the lead SNPs for *TortA* were not associated with CAD and myocardial infarction risk in the Coronary Artery Disease Genome-wide Replication and meta-analysis plus C4D consortium meta-analysis^29^ (Table IX in the online-only Data Supplement). Finally, the CAD-associated variants specifically in *COL4A1* from Coronary Artery Disease Genome-wide Replication and meta-analysis plus C4D were not associated with *TortA*, whereas CAD-associated *COL4A2* variants are only weakly associated with *TortA* (Table X in the online-only Data Supplement). Retinal arteriolar tortuosity traits have been previously associated with blood pressure,^10,12^ which may, therefore, link these variants with CAD; however, we found no evidence for an association between these lead variants and blood pressure in the International consortium for blood pressure GWAS analysis^41^ (Table XI in the online-only Data Supplement).

### Functional Annotation: *TortV*-Associated Variants

*ACTN4* encodes α-actinin 4 (alpha-actinin-4), a cross-linking protein belonging to the spectrin superfamily and mutations in this gene cause focal segmental glomerulosclerosis in humans. *ACTN2*, a homolog of *ACTN4*, interacts with *ACTN4* and missense mutations in *ACTN2* are linked to a range of cardiac diseases.^54^ Annotation by ENCODE^33^ indicates that the 2 genome-wide significant variants (rs1808382 and rs3786835) associated with *TortV* near *ACTN4* may have direct regulatory effects as they are located within a DNase I hypersensitivity site and in genomic regions enriched for promoter/enhancer histone marks in heart tissues (Table VI in the online-only Data Supplement). *ACTN4* and *CAPN12* (calcium-activated neural proteases 12) overlap by 339 bases at their 3′ ends and multitissue expression quantitative trait loci (eQTL) analysis confirms that these SNPs in *ACTN4* are associated with mRNA expression of both *ACTN4* and *CAPN12* in aorta, tibial artery, atrial appendage, and left ventricle of the heart (Table VII and Figure IX in the online-only Data Supplement). Additionally, this analysis indicates that the T allele at rs1808382 is correlated with lower *ACTN4* (artery aorta; *P*=2.1×10^−03^) and this correlation is even stronger with *CAPN12* (artery aorta; *P*=2.0×10^−07^). Whereas gene expression data using Genevestigator validated the high expression of *ACTN4* in arterial tissue, the highest expression of *CAPN12* appears to be in the hematopoietic system.

### Colocalization Analysis of SNPs in *ACTN4*/*CAPN12*

We further conducted eQTL/trait colocalization analyses with eCAVIAR^45^ and estimated the colocalization posterior probability for the lead variants that are associated with *TortV* and eQTLs. We found that the *CAPN12* has substantially higher colocalization posterior probability in heart left ventricle and artery tibial tissues than the *ACNT4* which indicates moderate colocalization between *CAPN12* eQTL and *TortV* (Table XIII in the online-only Data Supplement). The heart left ventricle is considered as the most relevant tissue (colocalization posterior probability; 0.38) for the *TortV*-associated lead variant (rs1808382)-*CAPN12* eQTL whereas for the other 3 tissues (heart atrial appendage; 0.22, artery aorta; 0.27, and artery tibial; 0.37) are considered as the relevant tissues for the second top variant (rs3786835) associated with *TortV*-*CAPN12* eQTL (Table XIII in the online-only Data Supplement).

### Association of *TortV*-Associated Variants With Cardiovascular Risk Factors

Lead SNPs in *ACTN4* were significantly associated with CAD in the Coronary Artery Disease Genome-wide Replication and meta-analysis plus C4D consortium meta-analysis^29^ (Table IX in the online-only Data Supplement) and were associated with CAD risk factors include HDL (high-density lipoprotein) cholesterol and triglycerides in the Global Lipid Genetics Consortium analysis^40^ but not associated with LDL (low-density lipoprotein) cholesterol in the Global Lipid Genetics Consortium analysis or blood pressure in the International Consortium for blood pressure GWAS analysis^41^ (Table XI in the online-only Data Supplement). Furthermore, we have confirmed the association between *TortV* and top variants in *ACTN4*/*CAPN12* in a sensitivity analysis that only included GoDARTS samples without any cardiovascular events before the retinal screening date (Table XII in the online-only Data Supplement). Moreover, a recent meta-analysis of 35 GWAS studies reported the association of SNP (rs11083475) in the *ACTN4* locus with increased resting heart rate,^42^ which may increase cardiovascular disease risk. This signal appears the same as that for *TortV* with strong linkage disequilibrium being observed between the lead SNPs for *TortV* and the index SNP for heart rate. Furthermore, we found that these SNPs were associated with heart rate in UK Biobank^43^ (Table XIV and Figure X in the online-only Data Supplement). Interestingly, *TortV*-associated SNPs also associated with AF in the recent AF GWAS analysis^44^ (Table XI in the online-only Data Supplement).

## Discussion

In this first GWAS meta-analysis for quantitative retinal vascular tortuosity traits, we identify a novel locus for retinal arteriolar tortuosity (*COL4A2*) and for retinal venular tortuosity (*ACTN4*/*CAPN12*), which were firmly established by replication in 3 independent cohorts. Power calculations indicated that a sample size of 4507 (stage 1 and stage 2), and 3094 (stage 1) and the effect size of 0.10 using Bonferroni correction (*P*<5×10^−8^) was adequate to provide 80% statistical power to detect the associations. Notably, we also identified in the discovery studies a genome-wide significant signal at a previously reported locus in/near *ATOH7*/*PBLD* for the optic disc radius and replicated previously identified variants for *CRVE* which validate our retinal traits measurement methods. However, we did not replicate the previously reported SNPs for *CRAE*, but the direction of the effect was consistent. This may due to differences in the phenotyping, smaller effect sizes for certain SNPs compared to the previous studies and need for more power to replicate this association (Table XV in the online-only Data Supplement).^15,16^

There are few limitations in this study. The meta-analysis of the lead SNPs for *TortA* and *TortV* in the replication cohorts (stage 2) lacks genome-wide significant *P* values which may due to the limited sample sizes. Other possible limitation of this study is that the retinal vascular traits were measured using different software in the discovery (eg, Vascular Assessment and Measurement Platform for Images of Retina) and replication cohorts (eg, SIVA). In spite of this limitation, our findings show consistent, homogeneous effects on tortuosity across 5 fairly diverse cohorts of European ancestry comprising individuals with and without diabetes. This highlights the robust nature of these genetic effects on retinal vascular topology. Also, these aspects strongly support the robustness our study design and findings.

Previous studies have reported association between *COL4A2* and CAD but the *TortA*-associated variants in *COL4A2* in the present study are not associated with cardiovascular disease and similarly *COL4A2* variants that are associated with CAD do not appear to be associated with arteriolar tortuosity suggesting that variants in this gene complex may be involved differentially in the pathophysiology of microvascular and macrovascular diseases. However, more work has to be done to determine the distinct role of genetic variants in *COL4A2*/*COL4A1* in different clinical conditions. In contrast, we found that retinal venular tortuosity-associated variants near *ACTN4*/*CAPN12* were associated with CAD, heart rate, and AF. Furthermore, the lead variant influences the expression of the *ACTN4*/*CAPN12* genes in the heart and blood vessel tissues. But our colocalization eQTL analysis found *CAPN12* as the most likely causal gene at the chromosome 19 locus. Our sensitivity analyses including samples without CAD before the date of acquisition of the measured retinal image indicates that the relationship between genetic predictors of retinal venular tortuosity and cardiovascular diseases is not due to reverse causation and demonstrate the robustness of our findings.

Notably, the *TortV*-associated variants have shared genetic architecture with other cardiovascular-related traits including HDL cholesterol and AF. A recent study reported the relationship between retinal venular tortuosity and lower HDL cholesterol in the Asian-based cross-sectional cohorts.^12^ Our in-silico look-ups indicate that the genetic determinants for *TortV* near *ACTN4* also associated with HDLC in the Global Lipid Genetics Consortium analysis GWAS but not associated with the LDLC which is consistent with the Asian-based study.^12^ A study from the ORCADES and Croatia-Korcula cohorts reported a weak association between retinal arteriolar tortuosity and systolic blood pressure and no significant association between retinal venular tortuosity trait and blood pressure.^10^ A recent larger epidemiological study in European-based prospective cohort reported an association with systolic blood pressure for *TortA*, and a weaker association for *TortV*.^55^ In this regard, neither the *TortA* nor *TortV*-associated variants were associated with systolic and diastolic blood pressure in the International Consortium for blood pressure GWAS analysis; therefore, it seems unlikely that observed associations with CAD or related traits are mediated through blood pressure.

In summary, this first GWAS for retinal arteriolar and venular tortuosity reveals associated SNPs of strong effects influencing the expression of *COL4A2* and *ACTN4*/*CAPN12*, respectively. Our results demonstrate that the *TortA*-associated variants in *COL4A2* are independent of CAD, myocardial infarction, and blood pressure, and point to a selective role of *COL4A2* rather than *COL4A1* in the retinal vessels. Strikingly, we found *TortV*-associated *ACTN4/CAPN12* SNPs are associated with CAD, HDL cholesterol, AF, and heart rate but not associated with blood pressure. Our findings appear to indicate *CAPN12* as the causal gene for *TortV* through colocalization analysis. However, detailed investigation and functional validation of this new finding are essential to elucidate the causal role of this locus and the relative contribution of *ACTN4* and *CAPN12* in the observed cardiovascular pathophysiology. These findings highlight the potential genetic impacts of retinal vasculature to provide new insights into cardiovascular disease.

## Acknowledgments

The study was designed by C.N.A. Palmer, A.S.F. Doney, and E. Trucco for the GoDARTS (Genetics of Diabetes Audit and Research in Tayside) cohort, J.F. Wilson for the ORCADES (Orkney Complex Disease Study) cohort, I.J. Deary for the LBC1936 (Lothian Birth Cohort 1936) cohort, O. Polasek for the Croatia-Split, and Croatia-Korcula cohorts. The Vascular Assessment and Measurement Platform for Images of Retina (VAMPIRE) software was developed by E. Trucco, T. MacGillivray, D. Relan, E. Brown, and B. Dhillon. Retinal images were collected, and analysis was performed by E.Trucco, T. MacGillivray, J.F. Wilson, L. Ballerini, M. Kirin, D. Relan, V. Vitart, S.S. Vaidya, and H. Campbell. Genotype data processing and statistical analysis were conducted by A. Veluchamy, K.E. Schraut, P.K. Joshi, L. Ballerini, M. Kirin, S. Harris, V. Vitart, C. Hayward, and K. Zhou. Bioinformatics analysis was performed by A. Veluchamy. The article was drafted by A. Veluchamy, C.N.A. Palmer, and A.S.F. Doney and revised by E. Trucco, J.F. Wilson, T. MacGillivray, I.J. Deary, S. Harris, E.R. Pearson, V. Vitart, C. Hayward, and K. Zhou. All the authors reviewed the article and approved the final version. We are grateful to all the participants in the GoDARTS study, the general practitioners, the Scottish School of Primary Care for their help in recruiting the participants, and to the whole team, which includes interviewers, computer and laboratory technicians, clerical workers, research scientists, volunteers, managers, receptionists, and nurses. The study complies with the Declaration of Helsinki. We acknowledge The National Institute for Health Research (NIHR) global health research unit on global diabetes outcomes research at the University of Dundee (INSPIRED project) Award number 16/136/102. We acknowledge the support of the Health Informatics Centre, University of Dundee for managing and supplying the anonymized data and NHS Tayside, the original data owner. We acknowledge the invaluable contributions of the research nurses in Orkney, the administrative team in Edinburgh and the people of Orkney. We thank the Lothian Birth Cohort 1936 (LBC1936) participants and team members who contributed to these studies. We acknowledge the staff of several institutions in Croatia that supported the field work, including but not limited to the University of Split and Zagreb Medical Schools and Croatian Institute for Public Health. Support from NHS Lothian R&D, and Edinburgh Imaging and the Edinburgh Clinical Research Facility at the University of Edinburgh is gratefully acknowledged.

## Sources of Funding

The Wellcome Trust United Kingdom Type 2 Diabetes Case-Control Collection (GoDARTS [Genetics of Diabetes Audit and Research in Tayside]) was funded by The Wellcome Trust (072960/Z/03/Z, 084726/Z/08/Z, 084727/Z/08/Z, 085475/Z/08/Z, and 085475/B/08/Z) and as part of the EU IMI-SUMMIT program. ORCADES (Orkney Complex Disease Study) was supported by the Chief Scientist Office of the Scottish Government (CZB/4/276 and CZB/4/710), the Royal Society, the MRC Human Genetics Unit, Arthritis Research UK and the European Union framework program 6 EUROSPAN project (contract no. LSHG-CT-2006-018947). DNA extractions were performed at the Wellcome Trust Clinical Research Facility in Edinburgh. Phenotype collection for LBC1936 was supported by Age UK (The Disconnected Mind project). Genotyping for LBC1936 was funded by the BBSRC (BB/F019394/1). The LBC1936 work was undertaken by The University of Edinburgh Centre for Cognitive Ageing and Cognitive Epidemiology, part of the cross council Lifelong Health and Wellbeing Initiative (MR/K026992/1); funding from the BBSRC and Medical Research Council (MRC) is gratefully acknowledged. The Croatia-Korčula and Croatia-Split study were funded by grants from the Medical Research Council (UK), European Commission Framework 6 project EUROSPAN (Contract No. LSHG-CT-2006-018947), European Commission Framework 7 project BBMRI-LPC (FP7 313010), the Republic of Croatia Ministry of Science, Education and Sports research grant (216-1080315-0302) and the Croatian Science Foundation (grant 8875). The SNP genotyping for the Korčula cohort was performed in Helmholtz Zentrum München, Neuherberg, Germany. Vascular Assessment and Measurement Platform for Images of Retina (VAMPIRE) team: Parts of the VAMPIRE software and its use for measuring the image set described here was funded by the Leverhulme Trust project RPG-419 Discovery of retinal biomarkers for genetics with large cross-linked data sets. VAMPIRE 3.1 has been further developed under funding from EPSRC (EPSRC EP/M005976/1) and the EU (REVAMMAD FP7-PEOPLE ITN, grant agreement 316990). For the analysis of the association of the identified genetic variants with heart rate, this research has been conducted using the UK Biobank Resource under Application Number 20405.

## Disclosures

I.J. Deary reports grants from Age UK, grants from Biotechnology and Biological Sciences Research Council and grants from the Medical Research Council during the conduct of the study. H. Campbell has received funding from EU IMI, Sanofi for work on pneumococcal disease, CRUK for cancer research, WHO and Gates foundation for research on child health during the conduct of the study. The other authors report no conflicts.

## Supplementary Material
