# Supplementary material for: Novel Genetic Locus Influencing Retinal Venular Tortuosity Is Also Associated With Risk of Coronary Artery Disease
Source: Arterioscler Thromb Vasc Biol. 2019 Oct 10;39(12):2542–52. doi: 10.1161/ATVBAHA.119.312552 (PMC6882544; doi:10.1161/ATVBAHA.119.312552)
Supplement: Supplementary file 1 [file atv-39-2542-s001.pdf]

Supplementary Materials

Novel locus influencing retinal venular tortuosity is also associated with risk of coronary artery disease

Abirami Veluchamy[1], Lucia Ballerini[2, 6], Veronique Vitart[3], Katharina E Schraut[4,5], Mirna Kirin[4,9], Harry Campbell[4], Peter K Joshi[4], Devanjali Relan[6,15], Sarah Harris[8,10,11], Ellie Brown[12] , Suraj S Vaidya[12], Baljean Dhillon[6], Kaixin Zhou[13], Ewan R Pearson[1], Caroline Hayward[3], Ozren Polasek[9], Ian J Deary[7,8], Thomas MacGillivray[6], James F Wilson[3,4], Emanuele Trucco[2], Colin NA Palmer[1*] , Alexander S F Doney[14*]

* These authors contributed equally to the study.

[1] Division of Population Health and Genomics, University of Dundee, Ninewells Hospital and Medical School, Dundee DD1 9SY - Scotland, United Kingdom.
[2] VAMPIRE project, Computer Vision and Image Processing Group, School of Science and Engineering (Computing), University of Dundee, Dundee, DD1 4HN - Scotland, United Kingdom.
[3] MRC Human Genetics Unit, MRC Institute of Genetics and Molecular Medicine, University of Edinburgh, Western General Hospital, Edinburgh, EH4 2XU - Scotland, United Kingdom.
[4] Centre for Global Health Research, Usher Institute of Population Health Sciences and Informatics, University of Edinburgh, Teviot Place, Edinburgh, EH8 9AG - Scotland, United Kingdom.
[5] Centre for Cardiovascular Science, Queen's Medical Research Institute, University of Edinburgh, Royal Infirmary of Edinburgh, 47 Little France Crescent, Edinburgh, EH16 4TJ - Scotland, United Kingdom.
[6] VAMPIRE project, Centre for Clinical Brain Sciences, Chancellor's Building, Royal Infirmary of Edinburgh, 49, Little France Crescent, Edinburgh, EH16 4SB – Scotland, United Kingdom.
[7] Psychology, University of Edinburgh, 7, George Square, EH8 9JZ - Edinburgh, United Kingdom.
[8] Centre for Cognitive Ageing and Cognitive Epidemiology, University of Edinburgh, 7, George Square, EH8 9JZ - Edinburgh, United Kingdom.
[9] Department of Public Health, University of Split , School of Medicine, Soltanska 2, 21000 Split ,Croatia.
[10] Medical Genetics Section, Centre for Genomic and Experimental Medicine and MRC Institute of Genetics and Molecular Medicine, University of Edinburgh ,Western General Hospital, Crewe Road, Edinburgh EH4 2XU, United Kingdom.
[11] Department of Psychology, University of Edinburgh, 7 George Square, Edinburgh EH8 9JZ, United Kingdom.
[12] Clinical Research Imaging Centre, Queen's Medical Research Institute, University of Edinburgh, Royal Infirmary of Edinburgh, 47 Little France Crescent, Edinburgh, EH16 4TJ – Scotland, United Kingdom.
[13]Renji Hospital, University of Chinese Academy of Sciences, Chongqing, 400062, China.
[14]Division of Molecular & Clinical Medicine, University of Dundee, Ninewells Hospital and Medical School, Dundee DD1 9SY - Scotland, United Kingdom.

[15] Department of Computer Science, BML Munjal University, Gurgaon, Haryana 122413, India.

Corresponding authors: Prof. Colin Palmer, Division of Population Health and Genomics, University of Dundee, Ninewells Hospital and Medical School, Dundee DD1 9SY - Scotland, United Kingdom, c.n.a.palmer@dundee.ac.uk, Telephone: +44 01382 383155 or Dr. Alex Doney, Division of Molecular & Clinical Medicine, University of Dundee, Ninewells Hospital and Medical School, Dundee DD1 9SY - Scotland, United Kingdom, a.doney@dundee.ac.uk.

Supplementary Note on Methods

Study participants

Discovery cohorts. GoDARTS comprises individuals of European-heritage from Tayside, Scotland who provided a sample of blood for genetic analysis and informed consent to link their genetic information to the anonymized electronic health records[1]. Approval for recruitment to GoDARTS was obtained from the Tayside Committee on Medical Research Ethics. 18,190 individuals were recruited with approximately half having type 2 diabetes at the time of recruitment with the other half being diabetes free. 7,290 individuals currently have genome-wide data for analysis. ORCADES is a family-based study of 2078 individuals aged 16-100 years recruited between 2005 and 2011 in the isolated Scottish archipelago of Orkney[2]. Genetic diversity in this population is decreased compared to Mainland Scotland, consistent with the high levels of endogamy historically. Fasting blood samples were collected and over 300 health-related phenotypes and environmental exposures were measured in each individual. All participants provided written informed consent and the study was approved by Research Ethics Committees in Orkney and Aberdeen.

Replication Cohorts. Lothian Birth Cohort 1936 (LBC1936) comprises 1091 participants who were born in 1936, most of whom took part in the Scottish Mental Survey of 1947[3]. The participants were recruited between 2004 and 2007 to a study to determine influences on cognitive aging. The fieldwork of Croatia-Korčula study was performed in 2007 in the eastern part of the island, targeting healthy volunteers who underwent complete eye examination and provided their blood sample for genetic analysis from the town of Korčula and the villages of Lumbarda, Žrnovo and Račišće. The Croatia-Split study included inhabitants of the Croatian coastal city of Split, aged 18 to 93. The sampling scheme was similar to Croatia- Korčula, and it took place during 2008 and 2009.

Retinal Vascular Parameters Measurement

Discovery cohorts. Standard digital retinal photographs used for routine diabetic retinopathy screening were obtained from the clinical record in 2,104 participants in GoDARTS. Images of the right eye of usable quality, defined using criteria reported in[4,5], were selected and categorized into two datasets based on the image pixel resolution: GoDARTS dataset 1 (n=788) and GoDARTS dataset 2 (n=1288). Standard fundal retinal photographs centred between the macula and optic disc were obtained using digital fundus camera from 1,743 participants in ORCADES among which 1595 individual's retinal images were selected after quality check. Retinal traits were quantified using VAMPIRE 3.1 for GoDARTS and ORCADES. VAMPIRE 3.1 (Vascular Assessment and Measurement Platform for Images of Retina) is used to measure the retinal vasculature from large numbers of images. The measurement process is organized as a sequence of automatic and manual stages. Manual stages allowed correction of errors made by the automatic software (e.g. vessel labeling as artery or vein) and to minimize their impact on statistical analysis. Briefly, after automatic detection of the optic disc and its radius

(*ODradius*), and establishment of the standard retina coordinates and zones (Figure I), the 6 thickest arterioles and 6 thickest venules appearing in a zone extending from the optic disc boundary to 2 optic disc diameters out was sampled. This was used to calculate the median (*TortA*) and maximum (*TortAmax*) arteriolar tortuosity and the median (*TortV*) and maximum (*TortVmax*) venular tortuosity. Central retinal arteriolar equivalent (*CRAE*), central retinal vein equivalent (*CRVE*) and Arteriole-to-Venule ratio (*AVR*) qualify vessel calibers and were measured in a zone 2 to 3 optic disc radii from the center of the optic disc.

Tortuosity is a quantitative measure of how much blood vessels twist and turn. Smaller values indicate straighter vessels. It has been implicated with various diseases, e.g. retinopathy of prematurity and other systemic diseases[6,7]. Several algorithms have been proposed to quantify tortuosity consistently with clinical grading; the algorithm used in VAMPIRE 3.1 was chosen based on the study by Lisowska *et al*.[8], which compared five classes of algorithms and provides an introduction to the topic. Results indicated that curvature-based algorithms performed best over a range of image characteristics, assuming high sampling rates of the vessel centre lines are available, which is the case in our work. Briefly, given the centre line of a vessel (defined as a segment of vein or artery between two consecutive junctions or the end of a retinal zone) the algorithm computes the total squared curvature divided by the segment's arc-length in pixels. Robust curvature estimates are provided by Annunziata's algorithm[9]. Tortuosity is dimensionless and therefore does not have units; the range of its values and scale depends on the algorithm adopted for its calculation. For comparisons between studies where different algorithms may have been used relative values (e.g. rank, as opposed to absolute values) or suitable normalizations are performed. In our case, venular tortuosity ranges from 2.51×10^{-6} to 1.27×10^{-3} and arterial tortuosity ranges from 1.45×10^{-6} to 1.55×10^{-3}. **Figure Ib.** shows an example of high venular and low arteriolar tortuosity vessels, with corresponding values computed by the VAMPIRE algorithm.

Replication Cohorts. Retinal fundus images using digital fundus camera from 1091 individuals from LBC1936 were collected at the recruitment stage and three years later, retinal traits were measured at a subsequent wave of testing using SIVA v3.1[10], at a mean age of 72.5 years (SD 0.7). Similarly, 897 and 976 individual's retinal fundus images centred between the macula and optic disc from Croatia- Korčula and Croatia-Split cohorts were collected using digital fundus camera and retinal traits were quantified using SIVA v3.1[10]. SIVA is a semi-automated software which can be used to measure the retinal vascular parameters including retinal vascular tortuosity and vascular caliber from retinal fundus images. After automatic detection of the optic disc, it placed a grid with reference to the center of the optic disc. Then the tortuous vessels were identified and tortuosity traits including *TortA*, and *TortV* were measured using the standard grading protocol by the software; this process was monitored by trained graders and adjusted manually if necessary.

Genotyping, quality control and imputation

Discovery cohorts. Quality control (QC) assessment were performed using PLINK1.9[11]. The poor quality variants, samples were excluded based on the quality control (QC) criteria included the following: SNPs call rate < 95%, Hardy–Weinberg equilibrium (HWE) P value < 10^{-6}, sample call rate < 95%, sample relatedness (IBD >0.8), and mismatch between reported and genotypic gender information. QC'd genotype data were imputed using IMPUTE2[12] on the basis of 1000 Genome Projects reference panel for all population. Finally, ancestry information of the individuals was derived using EIGENSTRAT[13] and first three principal components (PCs) were used for the association analyses to adjust the population stratification. ORCADES samples were genotyped with either the Illumina HumanHap300 bead chip (n=890) or the Illumina Omni1M (n=304) or Illumina Omni Express bead chips (n=1073). Alleles were called in Bead Studio/Genome Studio (Hap300/Omni) using Illumina cluster files. Subjects were excluded if they fulfilled any of the following criteria: genotypic call rate <98%, mismatch between reported and genotypic sex, unexpectedly low genomic sharing with first or second degree relatives, excess autosomal heterozygosity, and outliers identified by IBS clustering analysis. We excluded SNPs on the basis of minor allele frequency (<0.01/monomorphism), HWE ($P<10^{-6}$) and call rate (<97%). Given the very high overlap in SNPs between the two Omni chips, the intersection of QC'd SNPs was used to impute and phase individuals' genotyped on the Omni arrays together, whilst the Hap300 individuals were phased using SHAPEIT version 2[14] and imputed, separately. Imputation was carried out using IMPUTE2[12] and the 1,000 genomes reference panel. All ancestries phase1 integrated v3 reference panel, with a secondary reference panel of local exome sequences, sequenced using the Agilent Sure Select All Exon Kit v2.0. Illumina 100 bp paired end reads (average 30x depth), derived from 90 ORCADES subjects were chosen to optimally represent the haplotypes present. Imputations for the Hap300 and Omni subjects were then combined to form a combined panel of 37.5m SNPs for 2222 subjects[15].

Replication Cohorts. For LBC1936 individuals were excluded based on unresolved gender discrepancy, relatedness, call rate (≤0.95), and evidence of non-Caucasian descent. SNPs were included if they met the following conditions: call rate ≥ 0.98, minor allele frequency ≥ 0.01, and HWE test with P ≥ 0.001. Imputation to the 1000 Genomes Phase I v3 (March 2012 release) reference set was performed using minimac software[16]. For Croatia-Korčula and Croatia-Split samples and markers were excluded based on the following QC metrics; SNPs call rate < 98%, HWE with P value < 10^{-6}, sample call rate < 97%, MAF < 1%, outliers identified by IBS clustering analysis and unresolved gender discrepancy. Pre-phasing was performed using SHAPEIT version 2[14]. Imputation was carried out using IMPUTE2[12] software and 1000G Phase I v3 (March 14, 2012) reference panel.

Power calculation

The statistical power of detecting SNPs associations with the quantitative traits in two stage GWAS was calculated using the GWASPower/QT[17].

***In-silico* functional annotation**

Top SNPs were queried in the HaploReg v4.1[18] to catalogue the all SNPs near noncoding variants with $r^2 > 0.8$, and RegulomeDB[19] and GWAS catalog[20] databases used to explore the known and predicted regulatory elements and relevant genetic association studies. Functional effects of the top genes were predicted using the Encyclopedia of DNA Elements (ENCODE) project[21] and Roadmap Epigenomics projects, HaploReg, UCSC Genome Browser[22], and RegulomeDB. We used the expression Quantitative Trait Loci (eQTL) browser database in Genotype-Tissue Expression (GTEx)[23] to examine the cis-eQTLs for the top retinal traits associated SNPs mapped to the gene within the genomic region. Co-localization analysis was performed using eCAVIAR to investigate whether the same significant variant is causal in both GWAS of retinal venular tortuosity and gene expression in the relevant tissues and to identify causal gene[24]. We tested genes with significant cis-eQTL association (*ACTN4* and *CAPN12*) for the GWAS locus associated with *TortV* by analysing the lead variants using summary data from the present study and eQTL from GTEx v6. The co-localization posterior probability (CLPP) score was estimated for each variant in a GWAS locus for a given gene. Gene Visible web database from Genevestigator, open-access version which integrates manually curated gene expression data from microarray and RNAseq experiments. It was used to find the expression level of the genes in human tissues, cell lines, cancers or perturbations, associated with tortuosity traits. It is a large and growing reference database which integrates systematically annotated and manually curated gene expression data from high-quality microarray data (Affymetrix array) from several organisms[25].

***In-silico* look-ups of the novel variants for clinical outcomes**

We performed *in-silico* look-ups of variants of interest for cardiovascular related outcomes including coronary artery disease, myocardial infarction, hypertension, heart rate, HDLC, LDLC, Atrial fibrillation (AF) and triglycerides using summary results from different large-scale genome-wide studies on cardiovascular risk factors. The Coronary Artery Disease (CARDIoGRAMplus C4D) consortium[26] (CARDIoGRAMplusC4D) 1000 Genomes-based meta-analysis data comprised of 60,801 CAD cases and 123,504 controls from European, South Asian, and East Asian descent. In the Global Lipid Genetics Consortium, genetic data from 188,577 individuals of European, East Asian, South Asian, and African ancestry were used to examine the genetic loci associated with blood lipids levels. The International consortium for blood pressure[27] (ICBP) GWAS investigated the genetic loci associated with systolic and diastolic blood pressure traits in 200,000 individuals of European descent. A recent large-scale meta-analysis of GWAS includes 65,446 participants of multi-ethnic cohorts with AF identified several variants associated with AF.

UK Biobank data comprised of 112,008 participants who had a measure of pulse rate at the main interview and had genotype data[28]. We extracted the imputed genotypes for these SNPs from the interim release data set of the UK Biobank and performed multiple linear regressions including covariates of age, gender, and the first ten principal components obtained using EIGENSTRAT.

Web Resources

SNPTEST V 2.5.2, https://mathgen.stats.ox.ac.uk/genetics_software/snptest/snptest.html; **SHAPEIT v2**, https://mathgen.stats.ox.ac.uk/genetics_software/shapeit/shapeit.html; **IMPUTE v2** https://mathgen.stats.ox.ac.uk/impute/impute_v2.html; **1000 Genomes Project**, http://www.1000genomes.org/; **Vampire,** http://vampire.computing.dundee.ac.uk/index.html; **GWAMA** http://www.well.ox.ac.uk/gwama/; **R statistical program package**, http://www.r-project.org/; **BEDTools**, http://bedtools.readthedocs.org/en/latest/; **LocusZoom**, http://csg.sph.umich.edu/locuszoom/; **UCSC Genome Browser**, https://genome.ucsc.edu/; **HaploReg**, http://www.broadinstitute.org/mammals/haploreg/haploreg.php; **PLINK,** https://www.cog-genomics.org/plink2/; **Genotype-Tissue Expression (GTEx) project**, http://www.gtexportal.org/home/; **ENCODE**, http://www.genome.gov/encode/ and http://genome.ucsc.edu/ENCODE/; **RegulomeDB**, http://www.regulomedb.org/ ; **EIGENSTRAT**, http://genetics.med.harvard.edu/reich/Reich_Lab/Software.html; **Type2Diabetes Knowledge portal**, http://www.type2diabetesgenetics.org/ ; GWASpower/QT 1.0, http://www.mybiosoftware.com/gwaspowerqt-1-0-statistical-power-calculation-software-designed-gwas.html; **Genevisible**, https://genevisible.com/search; **GoDARTS**, http://diabetesgenetics.dundee.ac.uk/ ; **ORCADES**, http://www.orcades.ed.ac.uk/orcades/index.html ; **LBC1936**, http://www.lothianbirthcohort.ed.ac.uk/; **UK Biobank**, http://www.ukbiobank.ac.uk/.

Supplementary references

1. Hébert HL, Shepherd B, Milburn K, et al. Cohort Profile: Genetics of Diabetes Audit and Research in Tayside Scotland (GoDARTS). *Int J Epidemiol*. 2017:1-12.
2. McQuillan R, Leutenegger AL, Abdel-Rahman R, et al. Runs of Homozygosity in European Populations. *Am J Hum Genet*. 2008;83:359-372. doi:10.1016/j.ajhg.2008.08.007
3. Deary IJ, Gow AJ, Pattie A, Starr JM. Cohort profile: The lothian birth cohorts of 1921 and 1936. *Int J Epidemiol*. 2012;41:1576-1584. doi:10.1093/ije/dyr197
4. MacGillivray TJ, Cameron JR, Zhang Q, et al. Suitability of UK Biobank retinal images for automatic analysis of morphometric properties of the vasculature. *PLoS One*. 2015;10:1-10. doi:10.1371/journal.pone.0127914
5. Giachetti A, Trucco E. Accurate and reliable segmentation of the optic disc in digital fundus images the optic disc in digital fundus images. *J Med Imaging*. 2014;1. doi:0091-3286/2014/
6. Wilson CM, Wong K, Ng J, Cocker KD, Ells AL, Fielder AR. Digital image analysis in retinopathy of prematurity: A comparison of vessel selection methods. *J Am Assoc Pediatr Ophthalmol Strabismus*. 2012;16:223-228. doi:10.1016/j.jaapos.2011.11.015
7. MacGillivray TJ, Trucco E, Cameron JR, Dhillon B, Houston JG, Van Beek EJR. Retinal imaging as a source of biomarkers for diagnosis, characterization and prognosis of chronic

illness or long-term conditions. *Br J Radiol*. 2014;87:20130832. doi:10.1259/bjr.20130832
8. Lisowska A, Annunziata R, Loh GK, Karl D, Trucco E. An experimental assessment of five indices of retinal vessel tortuosity with the RET-TORT public dataset. *2014 36th Annu Int Conf IEEE Eng Med Biol Soc EMBC 2014*. 2014:5414-5417. doi:10.1109/EMBC.2014.6944850
9. Annunziata R, Kheirkhah A, Aggarwal S, Cavalcanti BM, Hamrah P, Trucco E. Tortuosity classification of corneal nerves images using a multiple-scale-multiple-window approach. 2017;44:113-120. doi:10.17077/omia.1016
10. Koh V, Cheung CYL, Zheng Y, Wong TY, Wong W, Aung T. Relationship of retinal vascular tortuosity with the neuroretinal rim: The singapore malay eye study. *Investig Ophthalmol Vis Sci*. 2010;51:3736-3741. doi:10.1167/iovs.09-5008
11. Chang CC, Chow CC, Tellier LC, et al. Second-generation PLINK: rising to the challenge of larger and richer datasets. *Gigascience*. 2015;4:7. doi:10.1186/s13742-015-0047-8
12. Marchini J, Howie B. Genotype imputation for genome-wide association studies. *Nat Rev Genet*. 2010;11:499-511. doi:10.1038/nrg2796
13. Price AL, Patterson NJ, Plenge RM, Weinblatt ME, Shadick N a, Reich D. Principal components analysis corrects for stratification in genome-wide association studies. *Nat Genet*. 2006;38:904-909. doi:10.1038/ng1847
14. O'Connell J, Gurdasani D, Delaneau O, et al. A General Approach for Haplotype Phasing across the Full Spectrum of Relatedness. *PLoS Genet*. 2014;10. doi:10.1371/journal.pgen.1004234
15. Joshi PK, Prendergast J, Fraser RM, et al. Local Exome Sequences Facilitate Imputation of Less Common Variants and Increase Power of Genome Wide Association Studies. *PLoS One*. 2013;8. doi:10.1371/journal.pone.0068604
16. Fuchsberger C, Abecasis GR, Hinds DA. Minimac2: Faster genotype imputation. *Bioinformatics*. 2015;31:782-784. doi:10.1093/bioinformatics/btu704
17. Feng S, Wang S, Chen CC, Lan L. GWAPower: A statistical power calculation software for genome-wide association studies with quantitative traits. *BMC Genet*. 2011;12:2-5. doi:10.1186/1471-2156-12-12
18. Ward LD, Kellis M. HaploReg: A resource for exploring chromatin states, conservation, and regulatory motif alterations within sets of genetically linked variants. *Nucleic Acids Res*. 2012;40:1-5. doi:10.1093/nar/gkr917
19. Boyle AP, Hong EL, Hariharan M, et al. Annotation of functional variation in personal genomes using RegulomeDB. *Genome Res*. 2012;22:1790-1797. doi:10.1101/gr.137323.112
20. Welter D, MacArthur J, Morales J, et al. The NHGRI GWAS Catalog, a curated resource of SNP-trait associations. *Nucleic Acids Res*. 2014;42:1001-1006. doi:10.1093/nar/gkt1229
21. Myers RM, Stamatoyannopoulos J, Snyder M, et al. A user's guide to the Encyclopedia of DNA elements (ENCODE). *PLoS Biol*. 2011;9. doi:10.1371/journal.pbio.1001046
22. Speir ML, Zweig AS, Rosenbloom KR, et al. The UCSC Genome Browser database: 2016 update. *Nucleic Acids Res*. 2015;44:D717-D725. doi:10.1093/nar/gkv1275
23. Ardlie KG, Deluca DS, Segre A V., et al. The Genotype-Tissue Expression (GTEx) pilot analysis: Multitissue gene regulation in humans. *Science (80-)*. 2015;348:648-660. doi:10.1126/science.1262110
24. Hormozdiari F, van de Bunt M, Segrè A V., et al. Colocalization of GWAS and eQTL Signals Detects Target Genes. *Am J Hum Genet*. 2016;99:1245-1260.

doi:10.1016/j.ajhg.2016.10.003
25. Hruz T, Laule O, Szabo G, et al. Genevestigator v3: a reference expression database for the meta-analysis of transcriptomes. *Adv Bioinformatics*. 2008;2008:420747. doi:10.1155/2008/420747
26. Schunkert H, Konig IR, Kathiresan S, et al. Large-scale association analysis identifies 13 new susceptibility loci for coronary artery disease. *Nat Genet*. 2011;43:333-338. doi:10.1038/ng.784\rng.784 [pii]
27. Ehret GB. Genome-Wide Association Studies: Contribution of Genomics to Understanding Blood Pressure and Essential Hypertension. *Curr Hypertens Rep*. 2011;12:17-25. doi:10.1007/s11906-009-0086-6.Genome-Wide
28. UK Biobank Coordinating Centre. UK Biobank: Protocol for a large-scale prospective epidemiological resource UK Biobank Coordinating Centre Stockport. 2007;06:1-112. doi:10.1126/science.311.5767.1535c

Supplementary Figures

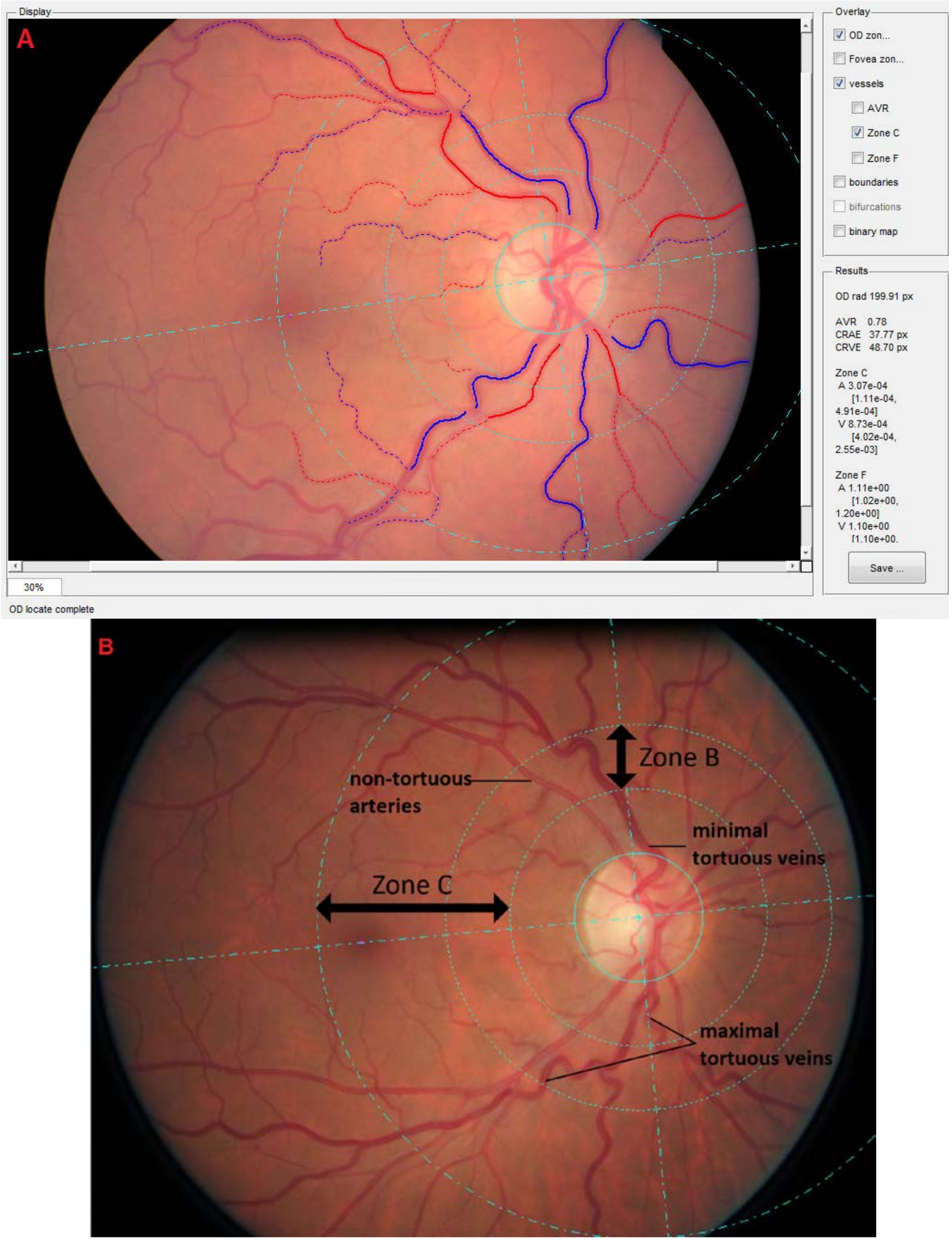

Figure I. Retinal fundus image. A) Solid lines (red for arterioles and dark blue for venules) represent the vessels detected automatically and measured by VAMPIRE (Vasculature Assessment and Measurement Platform for Images of the REtina) software (version 3.0, Universities of Edinburgh and Dundee, UK). Dotted lines (light blue) represent the measurement zones on a fundus image; based on optic disc (light blue circle) location and radius. B) Example of a retinal fundus image with tortuous veins ($1.27e^{-3}$), and non-tortuous arteries ($2.41e^{-5}$), with values computed by the algorithm; Zone B represents the ring 0.5-1 optic disc parameters away from the centre; Zone C represents the ring extending from optic disc boundary to 2 optic disc diameters away.

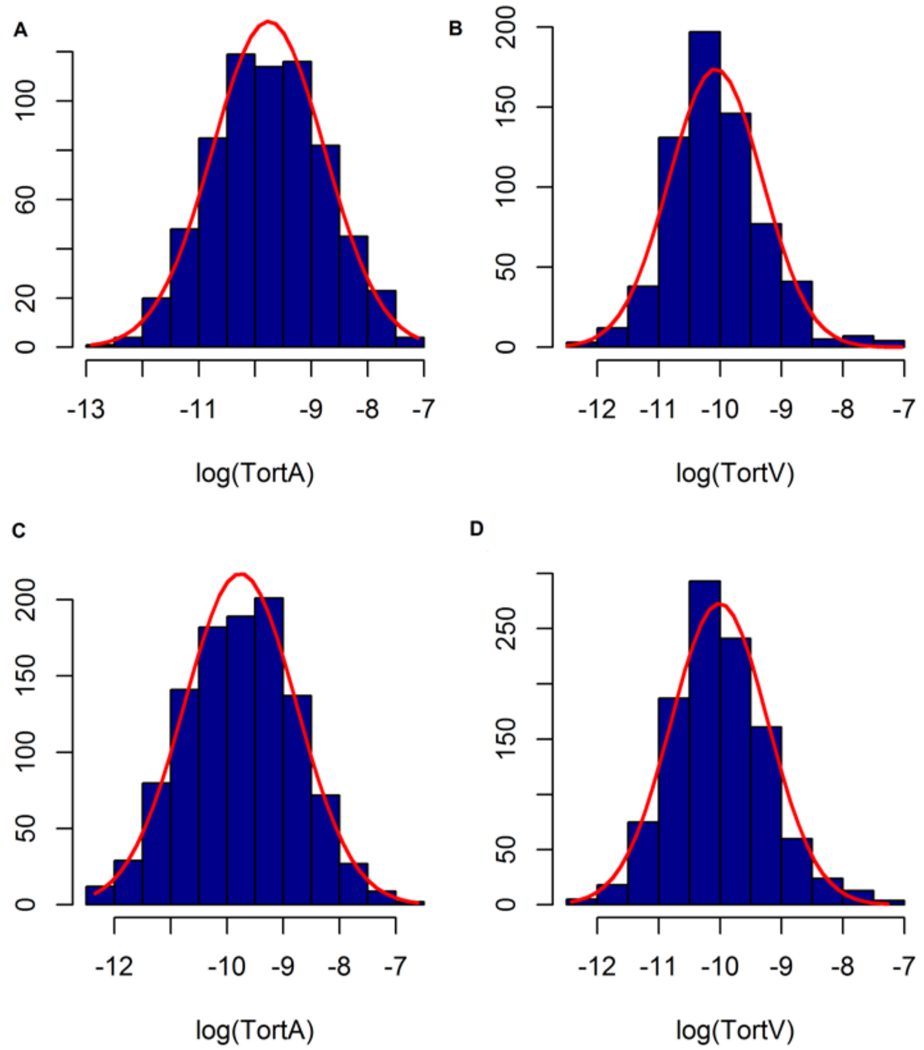

Figure II. Distributions of log transformed retinal tortuosity traits. A) log(*TortA*) GoDARTS data set 1 B) log(*TortV)* for GoDARTS data set 1 C) log(*TortA*) for GoDARTS data set 2 D) log(*TortV*) for GoDARTS data set 2.

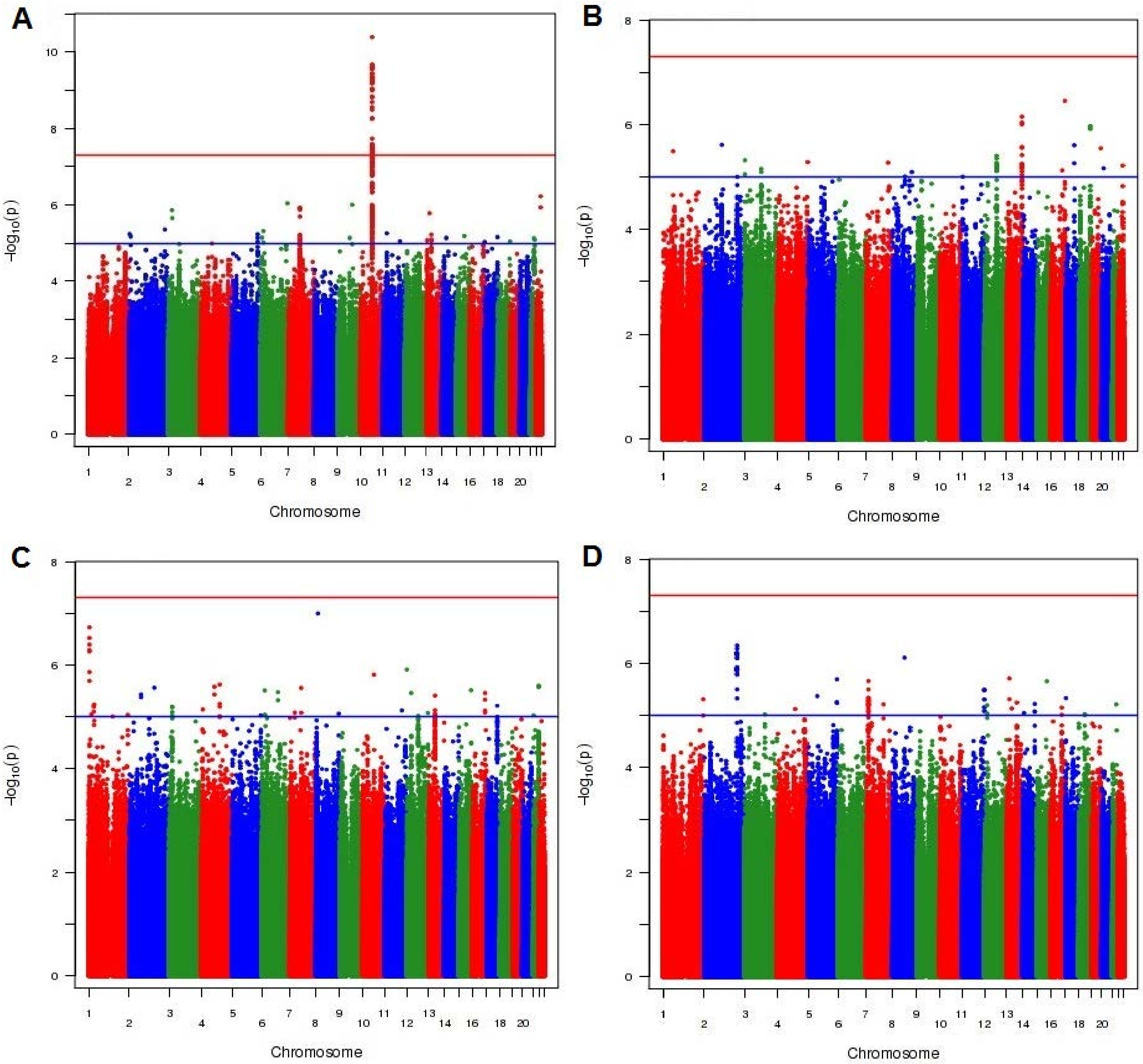
A
B
C
D
-log10(p)
Chromosome

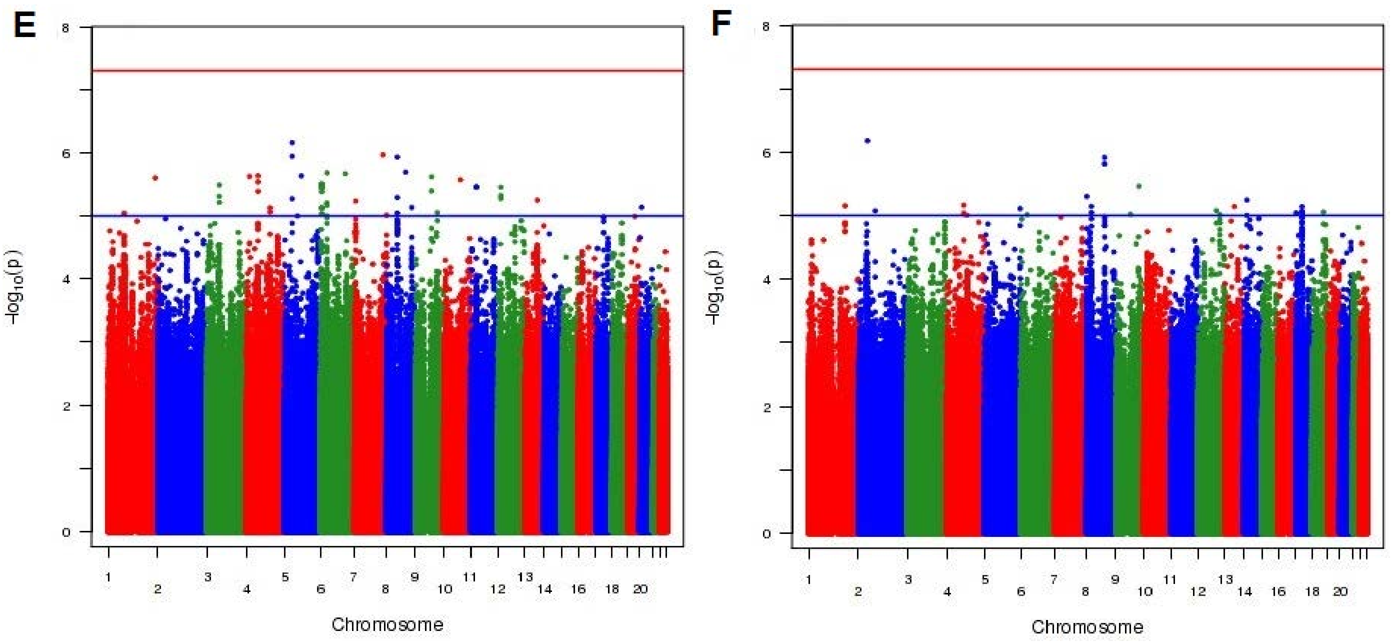

Figure III. Meta-analysis on genome-wide association results from two independent discovery cohorts. Manhattan plots for six quantitative retinal traits. A) represents the results from Optic Disc Radius (*ODradius*), B) represents the results from retinal arteriolar tortuosity maximum (*TortAmax*), C) represents the results from retinal venular tortuosity (*TortVmax*), D) represents the results from Central Retinal Arteriolar Equivalent (*CRAE*), E) represents the results from Central Retinal Venular Equivalent (*CRVE*), and F) represents the results from Arteriole-to-Venule ratio (*AVR*). The blue and red horizontal lines indicate the suggestive and genome-wide significance threshold ($P<5\times10^{-8}$), respectively.

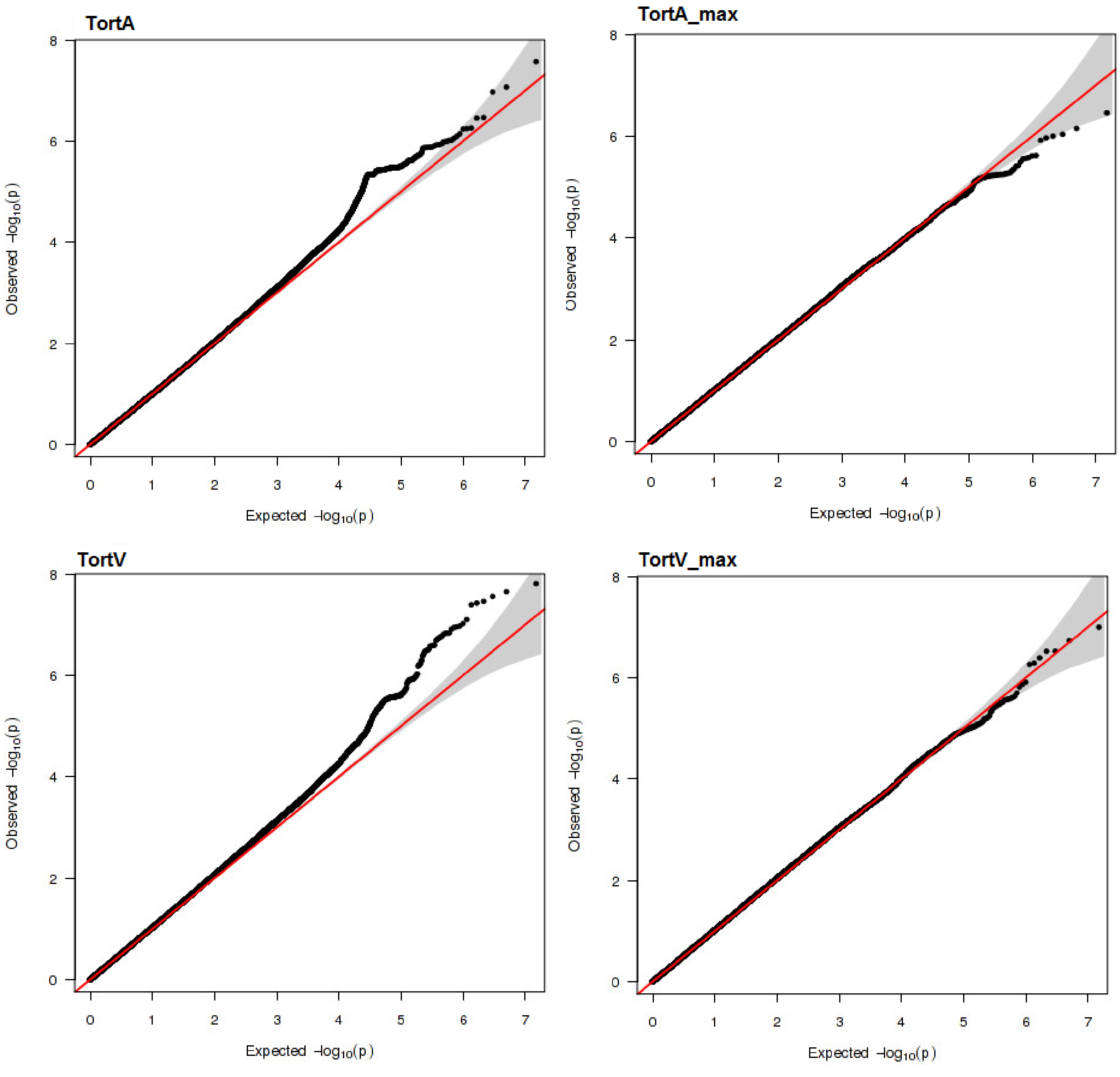
TortA
TortA_max
TortV
TortV_max
Observed −log10(p)
Expected −log10(p)
0 1 2 3 4 5 6 7
0 2 4 6 8

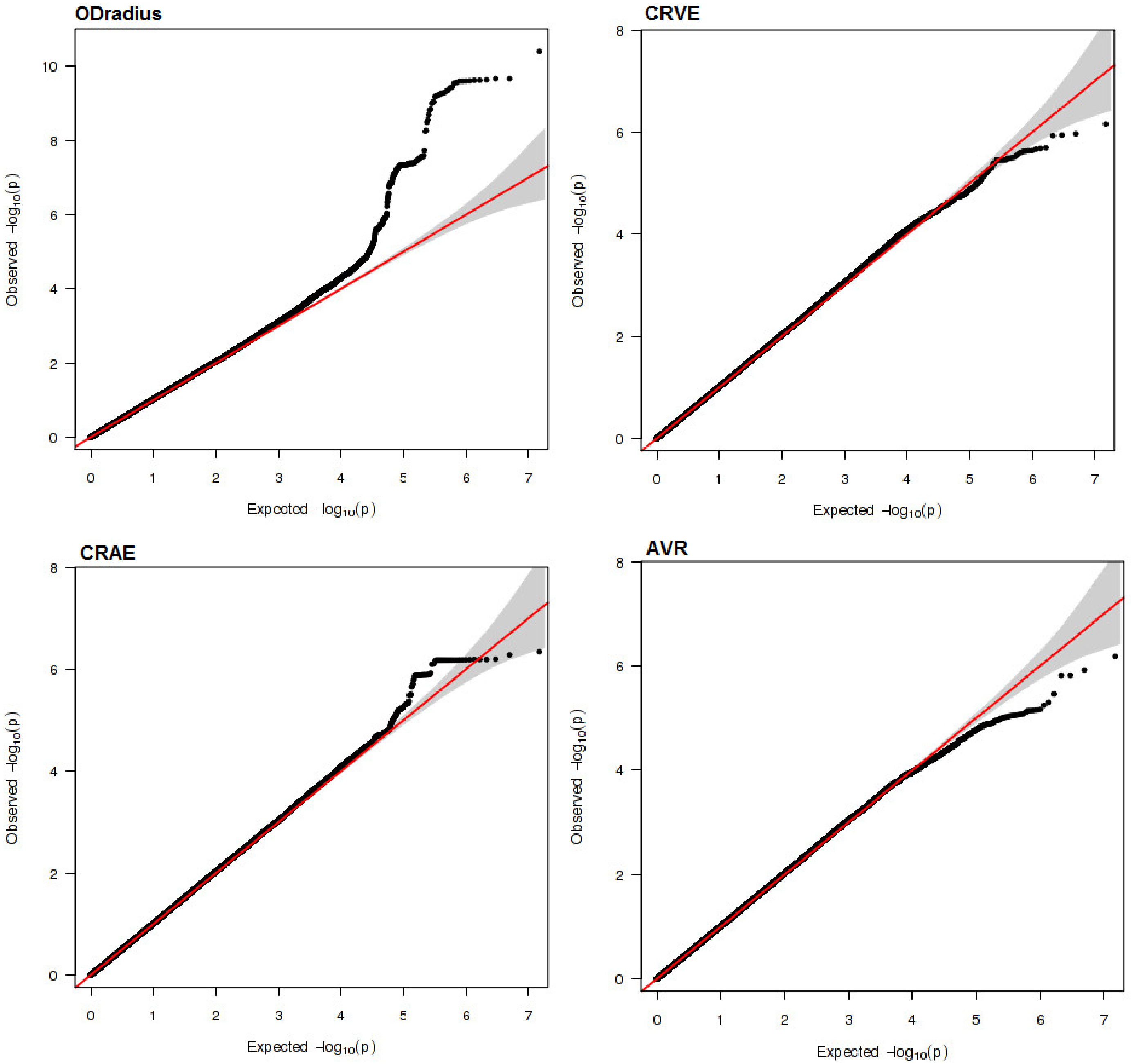

Figure IV. Quantile-quantile plots of GoDARTS-ORCADES meta-analysis for eight quantitative retinal blood vessel traits. Shaded areas represent 95% confidence intervals. Naturally log transformed -TortA: retinal arteriolar tortuosity, TortAmax: maximum retinal arteriolar tortuosity, TortV: retinal venular tortuosity, TortVmax: maximum retinal arteriolar tortuosity. ODradius: Optic Disc Radius, CRAE: Central Retinal Arteriolar Equivalent, CRVE: Central Retinal Venular Equivalent, AVR: Arteriole-to-Venule ratio.

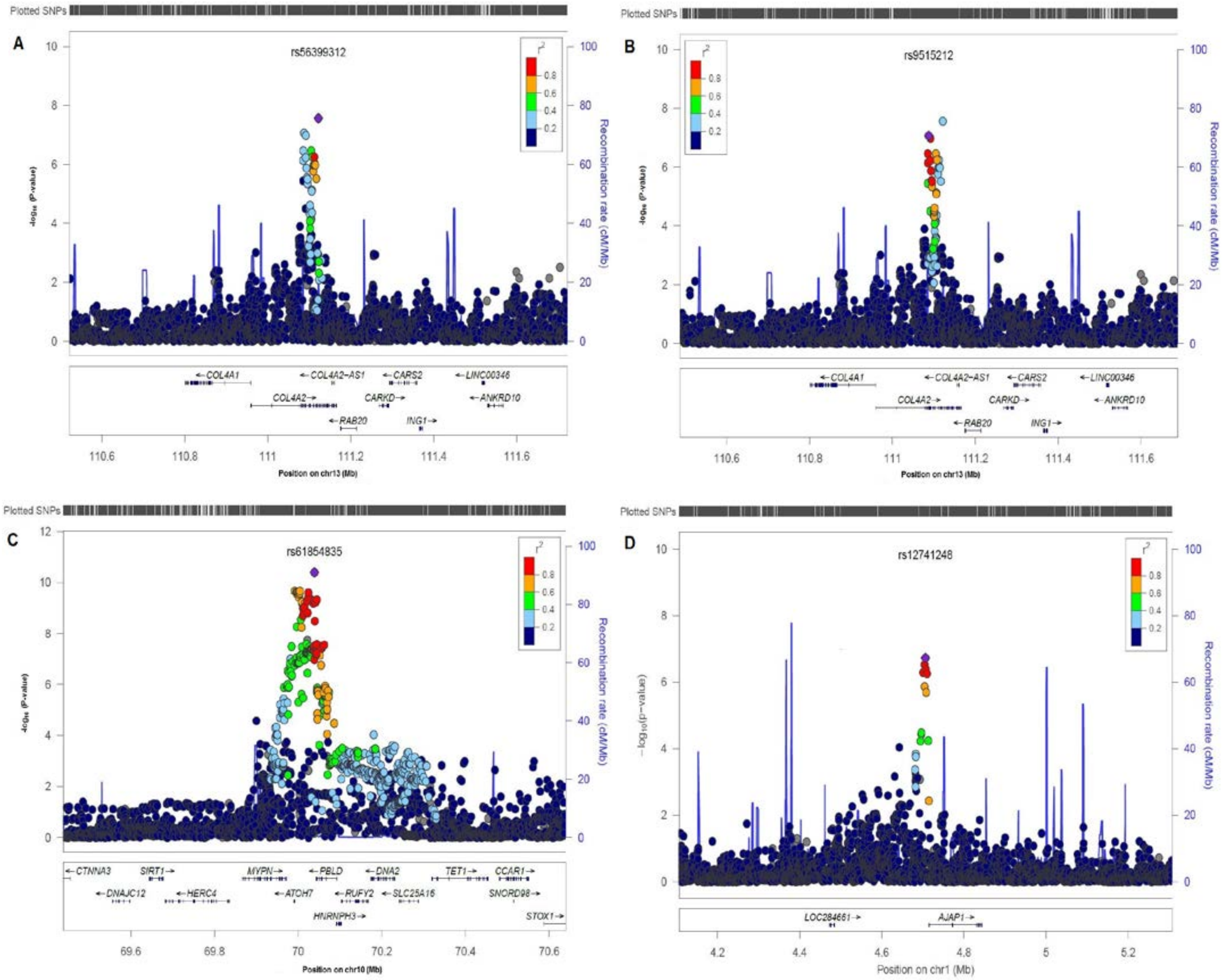

Figure V. Regional association plots of index SNP reached p-value < $5x10^{-7}$ in the meta-analysis of the two discovery study cohorts (GoDARTS and ORCADES). A) *TortA* B) maximum *TortA* C) *ODradius* D) maximum *TortV*.

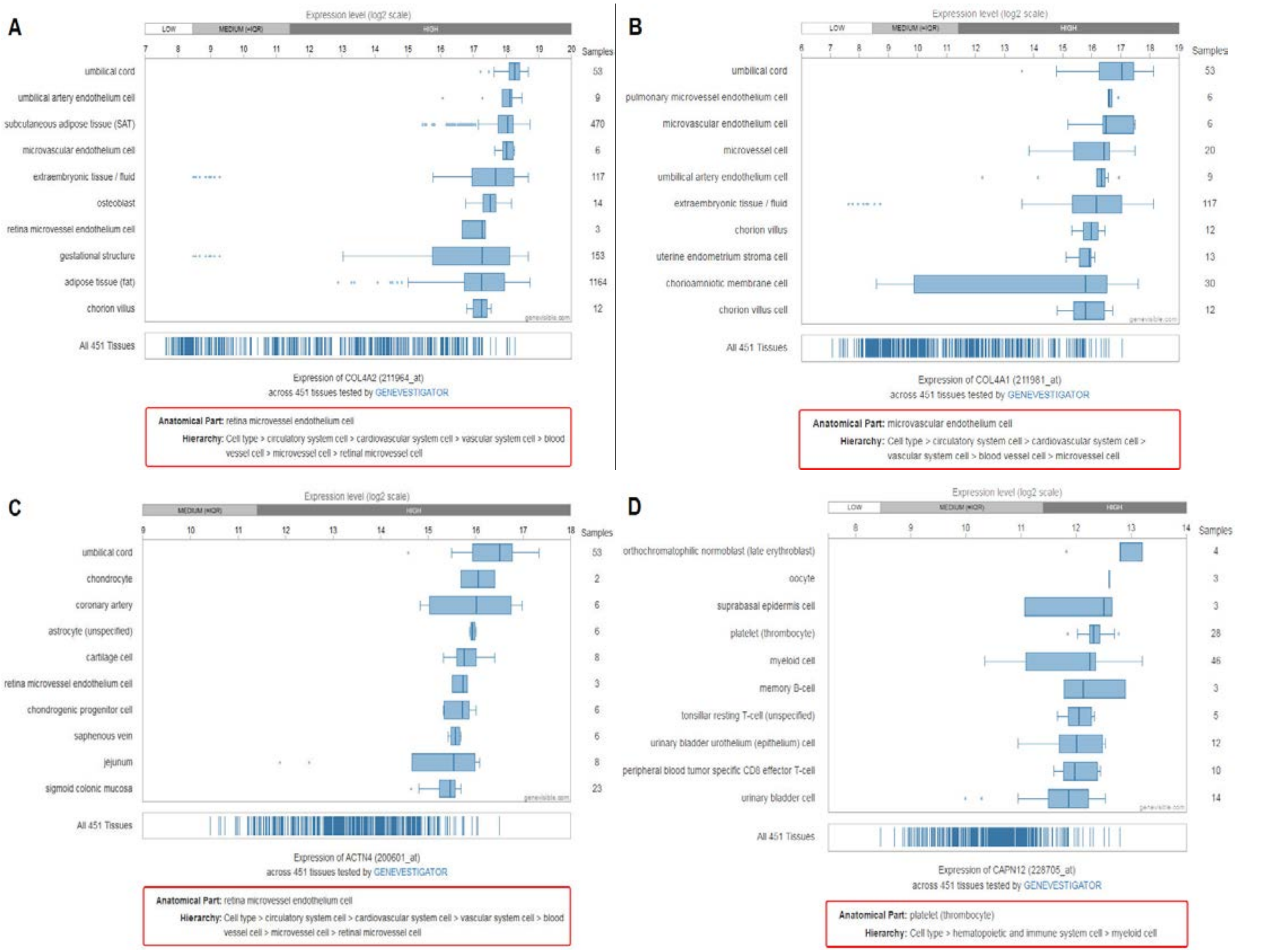

Figure VI. Box plots represent the expression level of genes associated with retinal blood vessel traits. A) *COL4A2* B) *COL4A1* C) *ACTN4* D) *CAPN12*. These plots were created using GENEVESTIGATOR which integrates manually curated gene expression data from microarray and RNAseq experiments. Blue lines at the bottom of the box indicates the gene expression level across 451 tissues in human. Y-axis depicts the top ten tissues and the sample size shown in secondary Y-axis. Colour scale (Low to High) at the top depicts the gene expression range in log2scale.

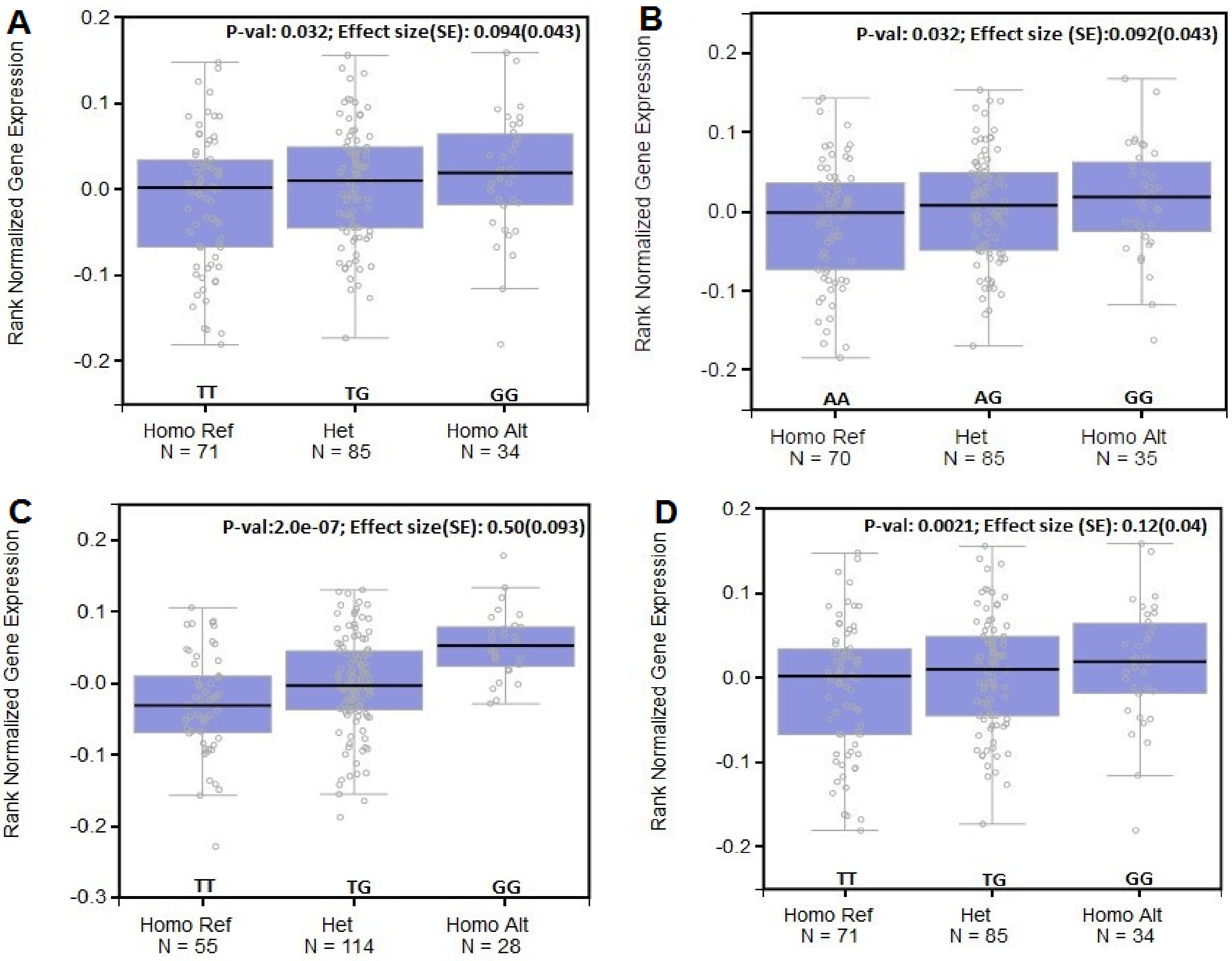

Figure VII. eQTL annotation of top GWAS variants for quantitative retinal vessel traits. Y-axis represents tissue-specific gene expression data which is normalized by rank normalization method while x-axis shows the GWAS lead variant genotypes. A) TortA associated SNP, rs7991229 is correlated with *COL4A2* expression in heart left ventricle; B) TortA associated SNP, rs9515212 is correlated with *COL4A2* expression in heart left ventricle tissue; C) TortV associated SNP, rs1808382 is correlated with *CAPN12* expression in artery aorta; D) TortV associated SNP, rs1808382 is correlated with *ACTN4* expression in artery aorta.

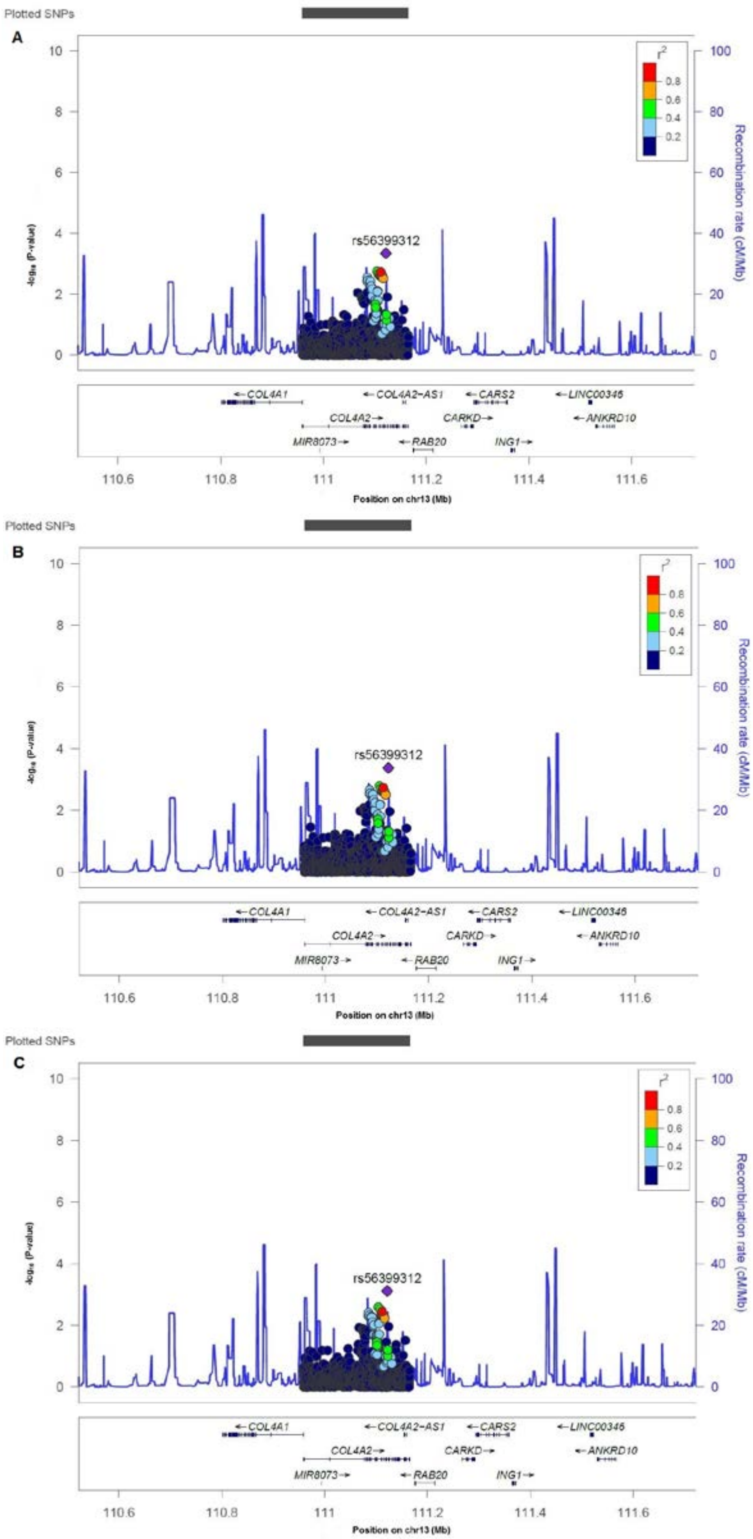

Figure VIII. Conditional analysis of the genome-wide significant variant (rs56399312) at *COL4A2* locus. Locus zoom plots for the *COL4A2* locus associated region (GoDARTS) conditioned on the CAD associated SNPs (rs11617955, rs4773144, rs9515203) reported previously in the GWAS study. A) Top SNP (TortA) Conditioned on rs11617955, B) Top SNP (TortA) Conditioned on rs4773144 C) Top SNP (TortA) Conditioned on rs9515203.

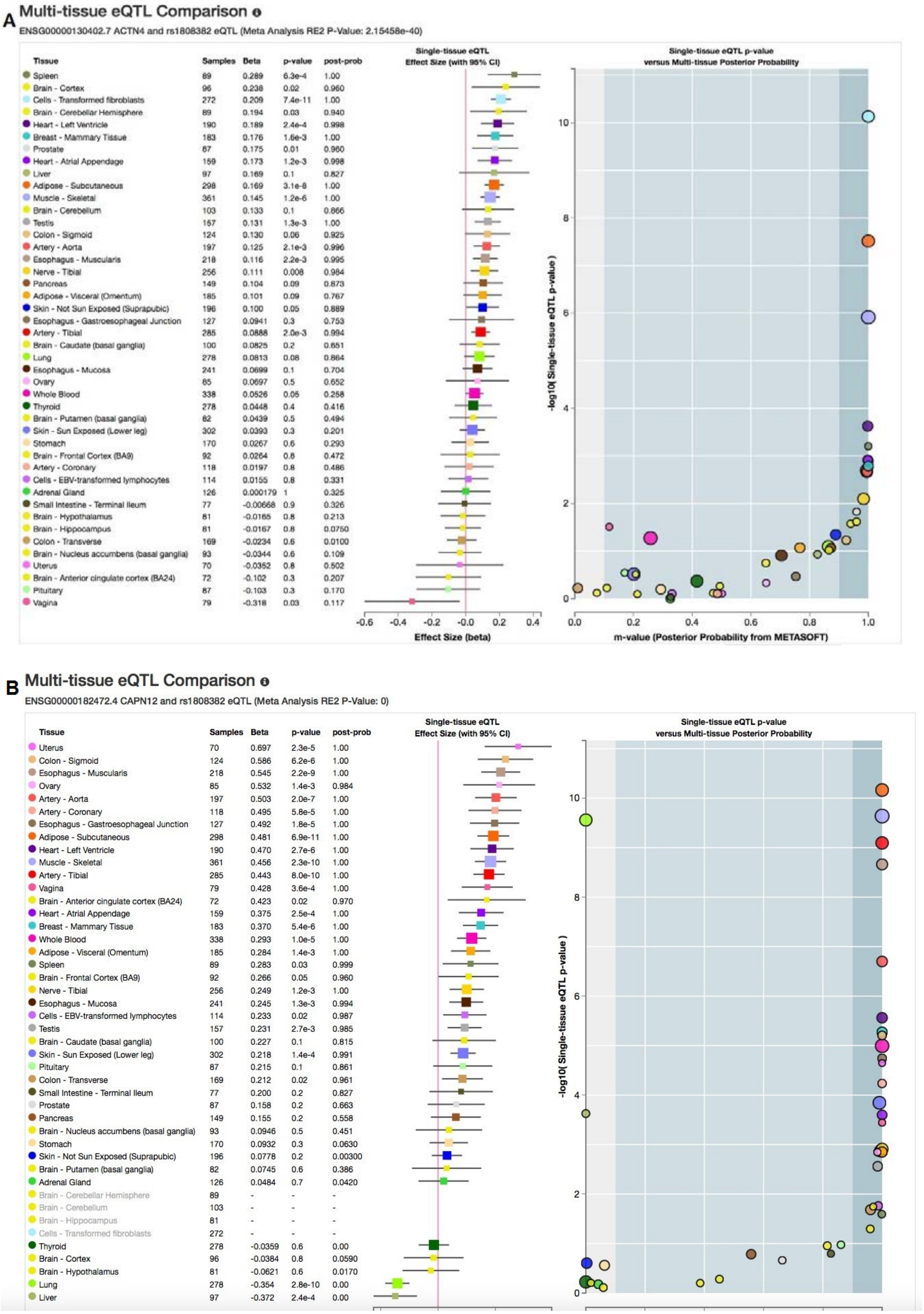

Figure IX. Multi-tissue eQTL comparison for TortV associated SNP, rs1808382 is correlated with A) *ACTN4* expression and B) *CAPN12* expression.

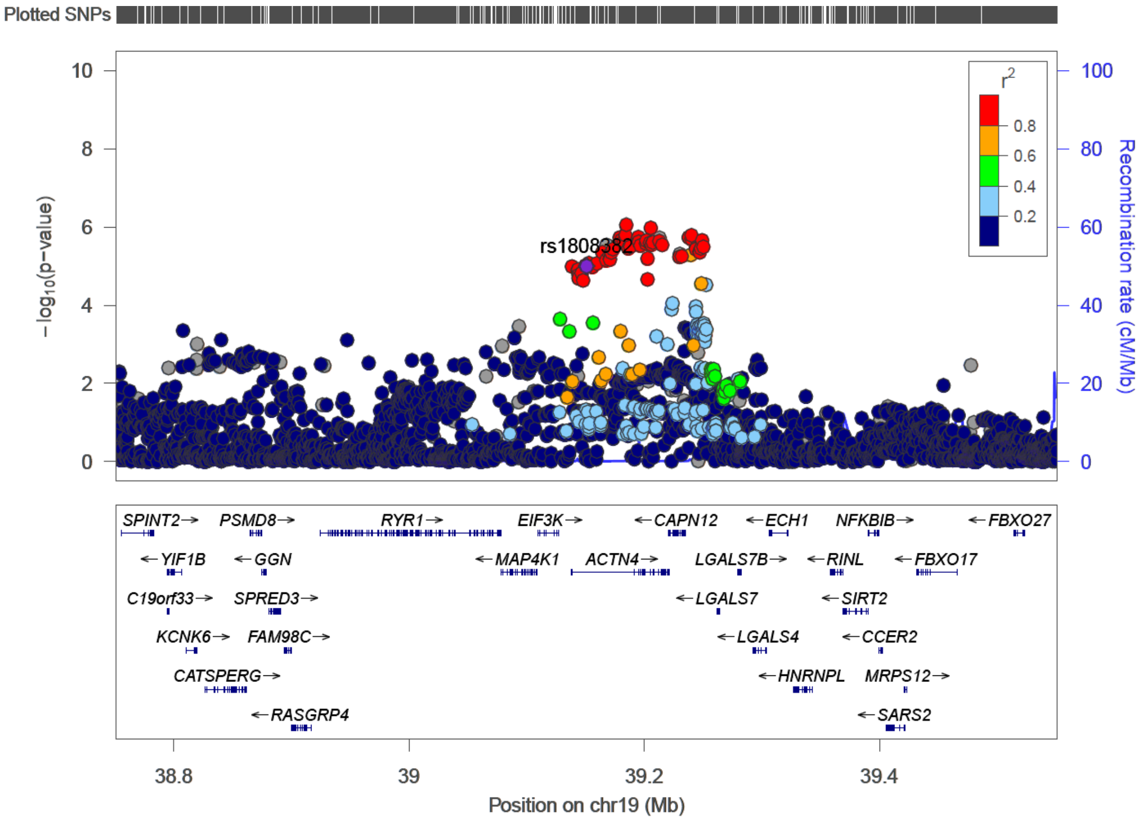

Figure X. Locus zoom plot for the *ACTN4* locus associated with TortV also associated with pulse rate in UK Biobank. The lead SNP, rs1808382 associated with TortV (Discovery and replication stage) in that region is indicated by purple colour solid diamond.

Table I. Descriptive statistics of variables for discovery study cohorts.

Traits	GoDARTS	ORCADES
Sample size	1742	1358
Age (yr.)	69.78 ± 9.63	52.23 ± 14.7
Male/Female	990/752	542/816
*ln*TortA (mean ± SD)	-9.76 ± 0.99	-10.94 ± 1.32
*ln*TortA max (mean ± SD)	-8.41± 0.92	-9.22 ± 1.17
*ln*TortV (mean ± SD)	-10.03± 0.78	-11.52 ± 1.01
*ln*TortV max (mean ± SD)	-8.66± 0.86	-9.76 ± 0.90
ODradius (mean ± SD)	208.56± 18.97	183.26 ± 19.46
CRAE (mean ± SD)	33.11± 2.39	29.09 ± 2.55
CRVE (mean ± SD)	43.74± 3.11	39.15 ± 3.62
AVR (mean ± SD)	0.76± 0.05	0.75 ± 0.08

Eight retinal vascular parameters are abbreviated as follows: Natural log transformed – Retinal Arteriolar Tortuosity (*ln*TortA), maximum TortA (*ln*TortAmax), Retinal Venular Tortuosity (*ln*TortV), and maximum TortV (lnTortV). Optic Disc radius (Odradius), Central Retinal Arteriolar Equivalent (CRAE), Arteriole-to-Venule ratio (AVR), Central Retinal Venular Equivalent (CRVE), SD, standard deviation. GoDARTS, Genetics of Diabetes Audit and Research in Tayside; ORCADES, Orkney Complex Disease Study.

Table II. Significant SNPs for each eight quantitative retinal blood vascular traits that reached $P<7x10^{-7}$ in the meta-analysis of discovery cohorts.

SNP	Chr.	Position	Gene	A1	A2	GoDARTS		ORCADES		Meta-analysis	
						BETA (SE)	P	BETA (SE)	P	BETA (SE)	P
**SNPs associated with arteriolar tortuosity (TortA)**											
rs56399312	13	111121981	*COL4A2*	T	C	0.186(0.037)	$6.67E^{-07}$	0.168(0.065)	0.0104734	0.182(0.032)	$2.70E^{-08}$
rs9515212	13	111087563	*COL4A2*	A	G	0.138(0.032)	$1.96E^{-05}$	0.188(0.055)	0.0006851	0.151(0.028)	$8.59E^{-08}$
rs7991229	13	111091995	*COL4A2*	T	G	0.136(0.032)	$2.30E^{-05}$	0.187(0.055)	0.0007278	0.149(0.027)	$1.07E^{-07}$
rs35131825	13	111104752	*COL4A2*	G	A	0.152(0.032)	$3.92E^{-06}$	0.134(0.059)	0.0254324	0.148(0.028)	$3.43E^{-07}$
rs6492273	13	111085494	*COL4A2*	T	C	-0.14(0.032)	$1.40E^{-05}$	-0.16(0.058)	0.0062893	-0.15(0.028)	$3.52E^{-07}$
rs9559803	13	111107863	*COL4A2*	A	G	0.150(0.032)	$5.66E^{-06}$	0.131(0.060)	0.0292305	0.146(0.029)	$5.58E^{-07}$
rs79749172	13	111112219	*COL4A2*	T	C	0.155(0.034)	$9.45E^{-06}$	0.152(0.063)	0.0175506	0.154(0.03)	$5.74E^{-07}$
rs9583493	13	111091632	*COL4A2*	C	A	0.137(0.032)	$2.23E^{-05}$	0.156(0.057)	0.0065341	0.141(0.028)	$5.83E^{-07}$
rs9559797	13	111085411	*COL4A2*	G	C	-0.14(0.032)	$2.29E^{-05}$	-0.153(0.06)	0.0082977	-0.14(0.028)	$7.26E^{-07}$
**SNPs associated with maximum – arteriolar tortuosity (TortA max)**											
rs1994653	16	86388330	*FOXF1-AS1*	C	G	-0.111(0.035)	0.001678	-0.26(0.056)	$3.98E^{-06}$	-0.15(0.029)	$3.52E^{-07}$
rs9515212	13	111087563	*COL4A2*	A	G	0.106(0.03)	0.000418	0.185(0.049)	0.0001794	0.127(0.025)	$7.08E^{-07}$
rs7991229	13	111091995	*COL4A2*	T	G	0.104(0.029)	0.000493	0.18(0.049)	0.0001962	0.125(0.025)	$9.19E^{-07}$
**SNPs associated with venular tortuosity (TortV)**											
rs1808382	19	39151034	*ACTN4*	G	T	-0.13(0.026)	$2.14E^{-06}$	-0.12(0.039)	0.0031218	-0.12(0.022)	$1.55E^{-08}$
rs3786835	19	39155880	*ACTN4*	G	A	-0.13(0.026)	$1.95E^{-06}$	-0.11(0.039)	0.0049245	-0.12(0.022)	$2.26E^{-08}$
rs8182572	19	39164188	*ACTN4*	T	C	-0.18(0.04)	$9.30E^{-06}$	-0.22(0.068)	0.0010322	-0.19(0.035)	$2.79E^{-08}$
rs62121814	19	39159729	*ACTN4*	C	T	-0.12(0.026)	$2.98E^{-06}$	-0.11(0.039)	0.0050112	-0.12(0.022)	$3.48E^{-08}$
rs16972767	19	39153044	*ACTN4*	G	A	-0.13(0.026)	$3.28E^{-06}$	-0.11(0.039)	0.0049088	-0.12(0.022)	$3.75E^{-08}$
rs73157566	12	129533847	*TMEM132D*	G	A	-0.29(0.061)	$3.28E^{-06}$	-0.31(0.113)	0.0054584	-0.29(0.054)	$4.07E^{-08}$

rs73157561	12	129517709	*TMEM132D*	A	C	-0.28(0.062)	6.14E-06	-0.31(0.113)	0.0056053	-0.29(0.054)	7.97E-08
rs979971	19	39144244	*ACTN4*	C	T	-0.11(0.025)	1.30E-05	-0.114(0.04)	0.0030991	-0.11(0.021)	9.60E-08
**SNPs associated with maximum – venular tortuosity (TortV max)**											
rs12741248	1	4706317	*AJAP1*	G	A	-0.25(0.051)	1.77E-06	-0.16(0.077)	0.0330366	-0.22(0.043)	1.87E-07
rs61764997	1	4704525	*AJAP1*	C	T	-0.24(0.052)	2.88E-06	-0.16(0.077)	0.0339291	-0.22(0.043)	3.01E-07
rs58047353	1	4704574	*AJAP1*	T	A	-0.24(0.052)	2.88E-06	-0.16(0.077)	0.0338477	-0.22(0.043)	3.02E-07
rs72857913	1	4707703	*AJAP1*	G	A	-0.24(0.052)	3.67E-06	-0.16(0.077)	0.0362085	-0.22(0.043)	4.08E-07
rs34719749	1	4700472	*AJAP1*	G	A	-0.25(0.052)	1.92E-06	-0.14(0.075)	0.0639505	-0.21(0.043)	5.18E-07
rs12071910	1	4710575	*AJAP1*	G	A	-0.24(0.052)	4.82E-06	-0.16(0.077)	0.0374194	-0.22(0.043)	5.53E-07
**SNPs associated with optic disc radius (ODradius)**											
rs61854835	10	70038894	*PBLD*	A	G	-3.46(0.724)	3.01E-06	-4.46(0.944)	2.25E-06	-3.84(0.575)	4.06E-11
rs7916697	10	69991853	*ATOH7*	A	G	3.00(0.681)	1.7 E-05	4.303(0.895)	1.52E-06	3.481(0.542)	2.18E-10
rs1900002	10	70004552	*ATOH7*	G	C	-3.12(0.689)	9.76E-06	-4.17(0.898)	3.51E-06	-3.51(0.547)	2.18E-10
rs7916410	10	69995667	*ATOH7*	T	C	3.042(0.683)	1.42E-05	4.25(0.898)	2.17E-06	3.487(0.544)	2.34E-10
rs1900005	10	69998055	*ATOH7*	A	C	3.039(0.683)	1.45E-05	4.248(0.897)	2.19E-06	3.483(0.544)	2.40E-10
rs4745950	10	69996455	*ATOH7*	A	G	3.04(0.683)	1.43E-05	4.247(0.897)	2.25E-06	3.484(0.544)	2.41E-10
rs4551651	10	70024529	*ATOH7*	A	G	-3.15(0.695)	9.90E-06	-4.26(0.921)	3.81E-06	-3.55(0.555)	2.49E-10
rs7897809	10	70000293	*ATOH7*	A	G	-3.06(0.684)	1.31E-05	-4.21(0.897)	2.71E-06	-3.48(0.544)	2.52E-10
rs1900004	10	70000881	*ATOH7*	C	T	-3.06(0.685)	1.28E-05	-4.19(0.896)	2.84E-06	-3.48(0.544)	2.54E-10
rs1900003	10	70004551	*ATOH7*	A	C	-3.11(0.690)	1.09E-05	-4.16(0.899)	3.66E-06	-3.51(0.548)	2.54E-10
rs4746743	10	69996292	*ATOH7*	G	A	3.006(0.683)	1.81E-05	4.27(0.898)	1.88E-06	3.472(0.544)	2.76E-10
rs9783176	10	70002393	*ATOH7*	A	T	-3.08(0.686)	1.19E-05	-4.15(0.896)	3.65E-06	-3.47(0.545)	2.86E-10
rs9988687	10	70024528	*PBLD*	A	G	-3.10(0.695)	1.38E-05	-4.26(0.923)	3.82E-06	-3.52(0.556)	3.65E-10
rs56238729	10	70001640	*ATOH7*	T	C	-3.07(0.685)	1.25E-05	-4.12(0.901)	4.89E-06	-3.45(0.546)	3.85E-10
rs10762199	10	70030464	*PBLD*	T	C	-2.96(0.680)	2.71E-05	-4.47(0.942)	2.08E-06	-3.48(0.552)	4.48E-10
rs4745957	10	70044702	*PBLD*	A	G	3.01(0.682)	1.72E-05	4.168(0.903)	3.93E-06	3.43(0.545)	4.74E-10

rs7912895	10	70021498	*PBLD*	A	G	-3.04(0.664)	8.22E^{-06}	-3.85(0.880)	1.21E^{-06}	-3.33(0.53)	5.13E^{-10}
rs12265247	10	70038398	*PBLD*	A	G	-2.99(0.678)	1.72E^{-05}	-4.14(0.902)	4.37E^{-06}	-3.41(0.542)	5.31E^{-10}
rs3740587	10	70020802	*PBLD*	G	A	3.04(0.664)	8.22E^{-06}	3.84(0.880)	1.27E^{-06}	3.331(0.53)	5.36E^{-10}
rs4746749	10	70009389	*ATOH7*	C	T	3.046(0.680)	1.25E^{-05}	4.14(0.902)	4.37E^{-06}	3.399(0.542)	5.79E^{-10}
rs4745956	10	70041583	*PBLD*	C	T	2.909(0.683)	3.28E^{-05}	4.26(0.897)	2.08E^{-06}	3.404(0.543)	5.97E^{-10}
rs4746748	10	70009388	*ATOH7*	G	A	3.04(0.680)	1.30E^{-05}	4.00(0.898)	8.29E^{-06}	3.393(0.542)	6.23E^{-10}
rs10998036	10	70016678	*ATOH7*	G	C	-2.97(0.681)	2.10E^{-05}	-4.31(0.932)	3.72E^{-06}	-3.44(0.55)	6.35E^{-10}
rs12246624	10	70036562	*PBLD*	C	T	-2.96(0.680)	2.19E^{-05}	-4.14(0.900)	4.25E^{-06}	-3.39(0.543)	6.64E^{-10}
rs61854803	10	70014638	*ATOH7*	T	C	-3.04(0.665)	8.10E^{-06}	-3.64(0.864)	2.53E^{-06}	-3.26(0.527)	9.12E^{-10}
rs10823166	10	70014140	*ATOH7*	T	C	-3.04(0.665)	8.18E^{-06}	-3.63(0.864)	2.57E^{-06}	-3.26(0.527)	9.29E^{-10}
rs3858145	10	70011838	*ATOH7*	A	G	-3.04(0.665)	8.03E^{-06}	-3.60(0.862)	2.91E^{-06}	-3.25(0.527)	1.01E^{-09}
rs3858146	10	70013250	*ATOH7*	C	T	-2.99(0.665)	1.12E^{-05}	-3.61(0.863)	2.89E^{-06}	-3.22(0.527)	1.45E^{-09}
rs10762198	10	70023053	*ATOH7*	A	G	2.89(0.663)	2.07E^{-05}	3.853(0.881)	1.22E^{-06}	3.24(0.53)	1.54E^{-09}
rs3858144	10	70011354	*ATOH7*	C	T	2.96(0.667)	1.46E^{-05}	3.64(0.874)	3.05E^{-06}	3.215(0.53)	2.05E^{-09}
rs61854799	10	70006659	*ATOH7*	T	C	-3.45(0.761)	9.85E^{-06}	-3.89(0.981)	7.31E^{-05}	-3.62(0.601)	2.79E^{-09}
rs10762201	10	70040111	*PBLD*	A	G	2.84(0.678)	4.34E^{-05}	3.94(0.899)	1.18E^{-05}	3.24(0.542)	3.26E^{-09}
rs4745951	10	69996624	*ATOH7*	A	G	3.26(0.743)	1.90E^{-05}	3.85(0.895)	6.92E^{-05}	3.48(0.59)	5.41E^{-09}
rs10823165	10	70008026	*ATOH7*	G	A	3.39(0.750)	1.02E^{-05}	3.67(0.971)	0.0001565	3.5(0.594)	5.72E^{-09}
rs4414109	10	70022176	*PBLD*	T	G	-4.07(0.933)	2.12E^{-05}	-4.46(1.218)	0.0002456	-4.22(0.741)	1.84E^{-08}
**SNPs associated with central retinal vein equivalent (CRVE)**											
rs62354706	5	38858653	*OSMR*	G	T	-1.39(0.299)	3.37E^{-06}	-0.824(0.41)	0.0432346	-1.19(0.241)	6.95E^{-07}
**SNPs associated with central retinal arteriolar equivalent (CRAE)**											
rs140656686	2	199134291	*PLCL1*	A	C	0.956(0.185)	3.39E^{-07}	0.37(0.279)	0.175775	0.779(0.154)	4.56E^{-07}
chr2:199134290: I	2	199134290	*PLCL1*	A	C	0.917(0.183)	6.94E^{-07}	0.373(0278)	0.179889	0.917(0.183)	5.25E^{-07}

rs77281498	2	199122864	*PLCL1*	G	T	0.881(0.169)	$2.45E^{-07}$	0.288(0.259)	0.267453	0.705(0.141)	$6.35E^{-07}$
**SNPs associated with arteriole-to-venule ratio (AVR)**											
rs6544787	2	45314906	*SIX2/SRBD1*	G	T	0.008(0.002)	$2.20E^{-05}$	0.008(0.003)	0.0102715	0.008(0.002)	$6.61E^{-07}$

Chromosome, base position and SNPID information is based on NCBI build 37 and dbSNP138. A2 is the effect allele for both cohorts. Het P (I^2) P value, I^2 for heterogeneity. Chr. Chromosome, BP base position, MAF minor allele frequency. Gene associated with trait reported previously are denoted in bold text. ODradius, Optic Disc Radius; CRAE, Central Retinal Arteriolar Equivalent; CRVE, Central Retinal Venular Equivalent; AVR, Arteriole-to-Venule ratio; Natural log transformed – TortA, retinal arteriolar tortuosity; TortAmax, maximum retinal arteriolar tortuosity; TortV, retinal venular tortuosity; TortVmax, maximum retinal venular tortuosity.

Table III. SNPs in *COL4A2* conditioned on top SNP rs56399312 (discovery cohorts), associated with *TortA*.

Conditional SNP	SNP (locus)	Meta-analysis			Conditional analysis			LD
		BETA	SE	P-value	BETA	SE	P-value	(R^2/D')
**Lead**	rs56399312 (13q34)	0.182	0.032	2.70E^{-08}	NA	NA	NA	NA
**rs56399312**	rs9515212 (13q34)	0.151	0.027	8.59E^{-08}	0.039	0.032	0.22061	0.3/0.64
**rs56399312**	rs7991229 (13q34)	0.149	0.027	1.07E^{-07}	0.037	0.0316	0.24363	0.31/0.64

TortA, retinal arteriolar tortuosity; LD, linkage disequilibrium; SE, standard error.

Table IV. Summary of previously reported significant SNPs associated with Optic Disc area, CRAE, and CRVE look-ups in GoDARTS-ORCADES meta-analysis study.

SNP (locus)	Candidate gene	Effect allele (Freq.)	GoDARTS & ORCADES Meta-analysis		Previously reported statistics		Study
			BETA (SE)	P-value	BETA (SE)	P-value	
**Optic Disc Size**							
rs12571093 (10q21)	*PBLD*	A (0.17)	-3.412(0.621)	**5.71×10^{-08}**	-0.291(0.046)	1.9×10^{-10}	
rs3858145 (10q21)	*ATOH7*	G (0.27)	-3.253(0.526)	**1.01×10^{-08}**	-0.227(0.036)	3.4×10^{-10}	
rs2241970(10q21)	*PBLD*	G (0.17)	-3.421(0.621)	**5.14×10^{-08}**	-0.233(0.042)	3.4×10^{-08}	
rs17231602(10q21)	*ATOH7*	A (0.15)	-3.078(0.655)	**3.39×10^{-06}**	-0.269(0.048)	$3.5x10^{-08}$	
rs7067601(10q21)	*PBLD*	T (0.85)	3.284(0.669)	**1.25×10^{-06}**	0.261(0.049)	$1.2\ x10^{-07}$	
rs10733843(10q21)	*PBLD*	G (0.85)	3.284(0.669)	**1.25×10^{-06}**	0.257(0.048)	$1.5\ x10^{-07}$	
rs4517412(10q21)	*PBLD*	G (0.83)	2.902(0.652)	**1.07×10^{-05}**	0.257(0.048)	$1.5\ x10^{-07}$	Stuart M *et*
rs6480314(10q21)	*MYPN*	A (0.82)	2.81(0.611)	**5.45×10^{-06}**	0.236(0.045)	$1.8\ x10^{-07}$	*al.*,2010
rs1192415(1p22)	*CDC7/TGFBR3*	A (0.82)	-1.92(0.614)	**0.002016**	-0.23(0.048)	$3.1\ x10^{-07}$	
rs491391(2q22)	*LRP1B*	C (0.05)	0.29(1.079)	0.791596	0.39(0.076)	$3.4\ x10^{-07}$	
rs1192404(1p22)	*CDC7/TGFBR3*	G (0.15)	1.67(0.653)	**0.011377**	0.24(0.047)	$4.6\ x10^{-07}$	
rs10762217(10q21)	*PBLD*	T (0.84)	2.956(0.654)	**7.80×10^{-06}**	0.241(0.048)	$4.9x10^{-07}$	
**Optic Disc Area**							Ramdas *et al.*,
rs1900004 (10q21)	*ATOH7/PBLD*	T (0.25)	-3.48(0.544)	**$2.54x10^{-10}$**	-0.070(0.006)	$2.7\ x10^{-35}$	2010
rs1192415(1p22)	*CDC7/TGFBR3*	A (0.82)	-1.917(0.613)	**0.002016**	-0.065(0.006)	$2.7\ x10^{-28}$	(Meta-analysis – 5
rs1362756(16q12.1)	*SALL1*	G (0.48)	-0.088(0.647)	0.893327	-0.033(0.005)	$5.07\ x10^{-09}$	cohorts)

**Optic Disc Area**							
rs6000762 (22q13)	*CARD10*	C (0.23)	0.967(0.567)	**0.091882**	0.045(0.007)	2.50 x10^{-11}	Khor *et al*., 2011 (two independent study cohorts)
rs9607469 (22q13)	*CARD10*	A (0.16)	0.667(0.649)	0.309612	0.051(0.007)	2.73 x10^{-12}	
rs6000764 (22q13)	*CARD10*	C (0.25)	1.241(0.551)	**0.025971**	0.04(0.007)	1.20 x10^{-09}	
rs6000766 (22q13)	*CARD10*	G (0.25)	1.260(0.558)	**0.025563**	0.04(0.007)	1.10 x10^{-09}	
rs8139526 (22q13)	*CARD10*	C (0.26)	1.092(0.552)	**0.05029**	0.037(0.006)	7.13 x10^{-09}	
**Optic Disc**							
rs1192419 (1p22)	*CDC7/TGFBR3*	G (0.82)	1.861(0.613)	**0.002698**	0.087(0.006)	7.98x10^{-56}	
rs6671926 (1q42)	*CDC42BPA*	A (0.07)	-3.88(0.924)	**3.2x10^{-05}**	−0.067(0.009)	3.69×10^{-14}	
rs1900004 (10q21)	*ATOH7*	T (0.25)	-3.48(0.544)	**2.54x10^{-10}**	−0.097(0.005)	1.13×10^{-73}	
rs1362756 (16q12)	*SALL1*	G (0.48)	-0.088(0.647)	0.893327	-0.033(0.005)	9.27×10^{-11}	
rs12406092 (1q24)	*F5*	A (0.29)	0.925(0.525)	**0.081513**	0.028(0.005)	3.32×10^{-09}	
rs1549733 (2q35)	*DIRC3*	T (0.21)	0.071(0.596)	0.906439	0.031(0.005)	4.03X10^{-09}	
rs11129176 (3p24)	*RARB*	A (0.28)	0.912(0.557)	0.105437	0.026(0.005)	1.74 X10^{-08}	Springelkamp *et al*., 2015 (Meta-analysis – 2 cohorts)
rs9860250 (3q12)	*ABI3BP*	G (0.16)	0.326(0.649)	0.618743	0.036(0.006)	2.42×10^{-09}	
rs11031436 (11p13)	*ELP4*	T (0.24)	0.144(0.564)	0.800193	0.033(0.005)	6.43×10^{-10}	
rs1511589 (12q21)	*TMTC2*	A (0.43)	-1.18(0.486)	**0.016323**	−0.028(0.005)	1.08×10^{-09}	
rs8034595 (15q26)	*NR2F2*	C (0.66)	-0.28(0.497)	0.576361	−0.026(0.005)	2.54×10^{-08}	
rs2412970 (22q12)	*HORMAD2*	G (0.44)	0.449(0.478)	0.35259	0.024(0.004)	3.40×10^{-08}	
rs9607469 (22q13)	*CARD10*	A (0.16)	0.667(0.649)	0.309612	0.041(0.006)	2.29×10^{-13}	
**CRVE**							
rs2287921 (19q13)	*RASIP1*	C (0.55)	-0.26(0.08)	**0.0015**	-2.1(0.20)	1.61x10^{-25}	
rs225717 (6q24)	*VTA1*	T (0.78)	-0.29(0.09)	**0.0017**	-1.9(0.23)	1.25x10^{-16}	Ikram *et al*., 2009
rs10774625 (12q24)	*ATXN2*	G (0.48)	0.13(0.08)	0.101	1.5(0.20)	2.15x10^{-13}	
rs17421627 (5q14)	*LINC00461*	G (0.06)	0.03(0.16)	0.878	3.0(0.37)	7.32x10^{-16}	
rs7824557 (8p23)	*LINC00529*	A (0.63)	0.04(0.08)	0.604	1.0(0.20)	3.80x10^{-7}	

**CRVE**								
rs7926971	*TEAD1*	G(0.45)	0.16(0.08)	**0.039837**	0.05(0.01)	3.1×10^{-11}	Jensen *et al.*, 2016
rs5442	*GNB3*	A(0.06)	-0.24(0.18)	0.183193	-0.11(0.02)	7.0×10^{-10}	
rs1800407	*OCA2*	T(0.08)	0.15(0.09)	0.313751	-0.11(0.02)	3.4×10^{-08}	
**CRAE**							
rs1800407	*OCA2*	T(0.08)	-0.11(0.11)	0.296258	-0.14(0.02)	6.5×10^{-12}	Jensen *et al.*, 2016
**CRAE**							
rs2194025 (5q14)	*TMEM16B /MEF2C*	C(0.09)	-0.14(0.111)	0.217	-1.60(0.23)	$2.11\text{x}10^{-12}$	Sim *et al.*, 2013
rs3744061 (17q25)	*SFRS2*	A (0.59)	-0.01(0.061)	0.969	-0.86(0.13)	$1.74\text{x}10^{-10}$	
rs2281827 (13q12.3)	*FLT1*	T (0.26)	0.019(0.07)	0.778	-0.70(0.16)	$1.44\text{x}10^{-5}$	

CRAE, Central Retinal Arteriolar Equivalent; CRVE, Central Retinal Venular Equivalent; and ODradius, Optic Disc Radius. Significant SNPs are bolded.

Table V. Descriptive statistics of variables for replication study cohorts.

Traits	LBC1936	Croatia-Korcula	Croatia-Split
Sample size	644	387	382
Age (yr.)	72.48 ± 0.72	54.28 ± 12.51	49.1 ± 14.23
Male/Female	334/310	122/265	154/228
TortA (mean ± SD)	-9.62 ± 0.235	-9.55 ± 0.26	-9.66 ± 0.25
TortV (mean ± SD)	-9.51 ± 0.242	-9.49± 0.21	-9.62 ± 0.22

Natural log transformed - Retinal Arteriolar Tortuosity (*ln*TortA), maximum TortA (*ln*TortAmax), Retinal Venular Tortuosity (*ln*TortV), and maximum TortV (lnTortV). SD, standard deviation. LBC1936, Lothian Birth Cohorts 1936; All Croatia, Croatia island of Korcula+Croatia Split.

Table VI. ***In-silico*** **functional annotation of significant SNPs for quantitative retinal vascular traits.**

Lead SNPs (locus)	LD >= 0.8; EUR	Nearest gene	Genome region	GWAS traits	Regulatory elements (RegulomeDB, UCSC genome browser, ENCODE, HaploReg)				
					**Transcription factor binding**	**Motifs**	**DNase / CpGs**	**Histone Marks Promoter**	**Histone Marks Enhancer**
**rs56399312 (13q34)**	2	*COL4A2*	intronic	*TortA*	Weak transcription/ minimal binding	HNF1 / TEF-1	Yes/	-	Yes
**rs9515212 (13q34)**	7	*COL4A2*	intronic	*TortA*	Unlikely to affect transcription factor binding	Yes	-	-	Yes
**rs7991229 (13q34)**	7	*COL4A2*	intronic	*TortA*	Unlikely to affect transcription factor binding	Yes	-	-	-
**rs1808382 (19q13.2)**	19	*ACTN4*	intronic	*TortV*	Weak transcription/ minimal binding evidence	Yes	Yes /No	Yes	Yes
**rs3786835 (19q13.2)**	38	*ACTN4*	intronic	*TortV*	*POLR2a* protein binding/minimal binding evidence	Yes	Yes /No	Yes	Yes
**rs73157566 (12q24.33)**	1	*TMEM132 D*	-	*TortV*	Weak transcription/ minimal binding evidence	Yes	Yes /Yes	-	-
**rs61854835 (10q21.3)**	2	*PBLD*	-	*ODradius*	Weak transcription/ minimal binding evidence	Yes	Yes /Yes	-	-
**rs7916697 (10q21.3)**	11	*ATOH7*	5'UTR	*ODradius*	TAF1 protein binding/minimal binding evidence	Yes	Yes/ Yes	Yes	Yes
**rs1900002 (10q21.3)**	18	*PBLD*	-	*ODradius*	Weak transcription/ minimal binding evidence	Yes	No/ No	No	Yes

Table VII. Significant top hits that reached P<1x10^{-7} for quantitative retinal vascular traits as eQTLs (GTEx) in different tissues.

Lead SNPs (locus)	Nearest gene	eQTL gene	eQTL ref. allele	eQTL P-value	eQTL Beta (SE)	Tissues	GWAS traits
**rs7991229 (13q34)**	*COL4A2*	*COL4A2*	T	0.032	0.094(0.043)<br>0.094(0.043)	Heart-Left Ventricle<br>Artery-Aorta	*TortA*
**rs9515212 (13q34)**	*COL4A2*	*COL4A2*	A	0.032	0.092(0.043)<br>0.094(0.042)	Heart-Left Ventricle<br>Artery-Aorta	*TortA*
**rs56399312**	*COL4A2*	*COL4A2*	T	0.064	0.095(0.051)	Heart-Left Ventricle	*TortA*
**(13q34)**	*COL4A2*	*COL4A2*		0.027	0.18(0.092)	Coronary Artery	
	COL4A2	*COL4A1*		0.048	0.20(0.087)	Coronary Artery	
**rs1808382 (19q13.2)**	*ACTN4*	*ACTN4*	G	0.0021	0.12(0.040)	Artery-Aorta	*TortV*
				0.0011	0.17(0.051)	Heart-Atrial appendage	
				0.0023	0.17(0.055)	Heart-Left Ventricle	
				$1.8E^{-10}$	0.45(0.092)	Artery-Tibial	
		CAPN12		$2.0e^{-07}$	0.50(0.093)	Artery-Aorta	
				0.0000032	0.46(0.095)	Heart-Left Ventricle	
**rs3786835 (19q13.2)**	*ACTN4*	*ACTN4*	G	0.0014	0.13(0.041)	Artery-Aorta	*TortV*
				0.0015	0.17(0.051)	Heart-Atrial appendage	
				0.0049	0.16(0.055)	Heart-Left Ventricle	
				0.0030	0.083(0.028)	Artery-Tibial	
				$5.1E^{-11}$	0.46(0.067)	Artery-Tibial	
		CAPN12		$2.9E^{-07}$	0.48(0.090)	Artery-Aorta	
				0.0000083	0.43(0.094)	Heart-Left Ventricle	

**rs61854835 (10q21.3)**	*PBLD*	*ATOH7*	A	0.0000021	-0.38(0.078)	Artery-Tibial	*ODradius*
				0.00050	-0.44(0.12)	Artery-Aorta	
		PBLD		0.000018	0.41(0.088)	Brain-Putamen (basal ganglia)	
**rs7916697 (10q21.3)**	*ATOH7*	*ATOH7*	A	4.3E^{-07}	0.37(0.071)	Artery-Tibial	*ODradius*
		PBLD		0.00011	-0.38(0.092)	Brain-Putamen (basal ganglia)	
**rs1900002 (10q21.3)**	*PBLD*	*ATOH7*	G	0.0000020	-0.36(0.074)	Artery-Tibial	*ODradius*
				0.0017	-0.36(0.11)	Artery-Aorta	
		PBLD		0.000054	0.23(0.056)	Artery-Aorta	
				0.00024	0.38(0.096)	Brain-Putamen (basal ganglia)	

Table VIII. Top SNPs in *COL4A2* associated with *TortA* conditioned on reported coronary artery disease SNPs.

SNP (locus)	BETA	SE	P-value	Condition analysis	LD(R^2/D')
**rs56399312 (13q34)**	0.182	0.032	2.70E-08	-	-
	0.172	0.037	0.00059	rs11617955,rs4773144, rs9515203	-
	0.188	0.037	0.00045	rs11617955 (*COL4A1*; 13q34)	0.001/0.063
	0.186	0.037	0.000415	rs4773144 (*COL4A2;* 13q34)	0.05/0.147
	0.174	0.037	0.000766	rs9515203 (*COL4A2*; 13q34)	0/0.10
**rs9515212 (13q34)**	0.151	0.027	8.59E-08	-	-
	0.126	0.032	0.003680	rs11617955,rs4773144, rs9515203	-
	0.136	0.032	0.003323	rs11617955 (*COL4A1*; 13q34)	0.003/0.184
	0.136	0.032	0.002749	rs4773144 (*COL4A2*; 13q34)	0.054/0.325
	0.126	0.032	0.004788	rs9515203 (*COL4A2*; 13q34)	0.044/0.301
**rs7991229 (13q34)**	0.149	0.027	1.07E-07	-	-
	0.123	0.032	0.004175	rs11617955,rs4773144, rs9515203	-
	0.134	0.032	0.003683	rs11617955 (*COL4A1*; 13q34)	-
	0.134	0.032	0.002981	rs4773144 (*COL4A2*; 13q34)	-
	0.124	0.032	0.005289	rs9515203 (*COL4A2*; 13q34)	-

Table IX. Summary of significant SNPs ($P<8x10^{-07}$) associated with tortuosity traits from discovery stage , replicated in myocardial infarction (MI) and coronary artery disease (CAD) GWAS.

SNP (locus)	Candidate gene	Effect allele (Freq.)	GoDARTS & ORCADES Meta-analysis		CARDioGramplus 1000G Additive model OCT 2015 - MI		CARDioGramplus 1000G Additive model OCT 2015 – CAD	
			BETA (SE)	P-value	BETA (SE)	P-value	BETA (SE)	P-value
TortA								
rs56399312	*COL4A2*	C (0.269)	0.182(0.032)	$2.70E^{-08}$	0.001(0.013)	0.925632	0.008(0.012)	0.489081
rs9515212	*COL4A2*	G (0.42)	0.151(0.028)	$8.59E^{-08}$	0.007(0.010)	0.500195	0.01(0.009)	0.3130185
rs7991229	*COL4A2*	G (0.42)	0.149(0.027)	$1.07E^{-07}$	0.007(0.010)	0.463883	0.009(0.009)	0.3129999
rs35131825	*COL4A2*	A (0.37)	0.148(0.028)	$3.43E^{-07}$	0.013(0.011)	0.224723	0.019(0.01)	0.0534524
rs6492273	*COL4A2*	C (0.59)	-0.146(0.03)	$3.52E^{-07}$	-0.005(0.01)	0.643380	-0.008(0.01)	0.3840433
rs9559803	*COL4A2*	G (0.37)	0.146(0.029)	$5.58E^{-07}$	0.013(0.011)	0.237036	0.016(0.009)	0.0970838
rs79749172	*COL4A2*	C (0.28)	0.154(0.03)	$5.74E^{-07}$	0.005(0.012)	0.699773	0.007(0.011)	0.4807673
rs9583493	*COL4A2*	A (0.41)	0.141(0.028)	$5.83E^{-07}$	0.007(0.010)	0.453616	0.009(0.009)	0.3115589
rs9559797	*COL4A2*	C (0.58)	-0.142(0.03)	$7.26E^{-07}$	-0.008(0.01)	0.418938	-0.012(0.01)	0.2187231
TortV								
rs1808382	*ACTN4*	T (0.475)	-0.123(0.022)	$1.55E^{-08}$	-0.008(0.01)	0.437167	-0.023(0.009)	**0.0154457**
rs3786835	*ACTN4*	A (0.471)	-0.121(0.022)	$2.26E^{-08}$	-0.007(0.01)	0.459396	-0.021(0.009)	**0.0224289**
rs8182572	*ACTN4*	C (0.16)	-0.193(0.035)	$2.79E^{-08}$	0.001(0.019)	0.975824	-0.023(.0172)	0.1798734
rs62121814	*ACTN4*	T (0.472)	-0.119(0.021)	$3.48E^{-08}$	-0.006(0.01)	0.537906	-0.019(0.009)	**0.0340804**
rs16972767	*ACTN4*	A (0.471)	-0.119(0.021)	$3.75E^{-08}$	-0.007(0.01)	0.522132	-0.021(0.009)	**0.0261441**
rs73157566	*TMEM132D*	A (0.043)	-0.294(0.054)	$4.07E^{-08}$	-0.088(0.033)	**0.008094**	-0.046(0.030)	0.1243125
rs73157561	*TMEM132D*	C (0.043)	-0.292(0.054)	$7.97E^{-08}$	-0.095(0.035)	**0.007413**	-0.059(0.032)	0.0687363
rs979971	*ACTN4*	T (0.48)	-0.113(0.021)	$9.60E^{-08}$	-0.006(0.01)	0.527511	-0.021(0.009)	**0 .02573**

CARDioGramplus, Coronary ARtery DIsease Genome wide Replication and Meta-analysis (CARDIoGRAM) plus The Coronary Artery Disease (C4D) Genetics. Significant SNPs are bolded.

Table X. Summary of previously reported significant SNPs associated with coronary artery disease (CARDioGramplus C4D) look-ups in GoDARTS-ORCADES meta-analysis study for retinal arteriolar tortuosity trait.

SNP (locus)	Candidate gene	CARDioGramplus 1000G Additive model OCT 2015 – CAD		GoDARTS & ORCADES Meta-analysis - *TortA*	
		**BETA (SE)**	**P-value**	**BETA (SE)**	**P-value**
rs11617955	*COL4A1*	-0.089(0.016)	3.55×10^{-08}	-0.025(0.047)	0.599638
rs4773144	*COL4A2*	-0.052(0.010)	3.87×10^{-07}	-0.021(0.087)	0.22419
rs9515203	*COL4A2*	0.071(0.012)	9.33×10^{-10}	0.087(0.031)	**0.005522**
rs55940034	*COL4A2*	-0.067(0.011)	3.49×10^{-10}	-0.084(0.031)	**0.007359**
rs9515201	*COL4A2*	0.064(0.010)	5.46×10^{-10}	0.075(0.029)	**0.012811**
rs11838776	*COL4A2*	-0.068(0.011)	1.83×10^{-10}	-0.083(0.031)	**0.008251**
rs7986871	*COL4A2*	0.052(0.010)	5.76×10^{-07}	0.033(0.027)	0.242749
rs4773143	*COL4A2*	0.052(0.010)	4.56×10^{-07}	0.034(0.027)	0.21799
rs4773141	*COL4A1*	0.071(0.012)	2.14×10^{-09}	0.023(0.031)	0.450293
rs12866570	*COL4A1*	-0.085(0.012)	7.86×10^{-08}	-0.066(0.048)	0.174417

CARDioGramplus, Coronary ARtery DIsease Genome wide Replication and Meta-analysis (CARDIoGRAM) plus The Coronary Artery Disease (C4D) Genetics. Significant SNPs are bolded.

Table XI. Genome-wide significant SNPs ($P<8x10^{-07}$) for retinal tortuosity traits from discovery stage, are associated with different traits (Type2Diabetes Knowledge Portal, cardiovascular disease knowledge portal and ICBP).

SNP	Candidate gene	Effect allele (Freq.)	GoDARTS & ORCADES Meta-analysis		Trait	Beta / P-value	GWAS Data set
			BETA (SE)	P-value			
rs7991229	*COL4A2*	G (0.42)	0.136(0.03)	$1.07E^{-07}$	Systolic and	0.162/0.116	ICBP
					Diastolic blood pressure	0.058/0.373	ICBP
					HDLC	0.005/0.292	GLGC
					LDLC	0.006/0.214	GLGC
					Triglycerides	0.002/0.972	GLGC
					Pericardial adipose tissue volume	NA/**0.03**	VATGen GWAS
					Coronary artery disease	0.01/0.313	CARDIoGRAMplus C4D
					Heart rate	-0.003/0.952	Heart rate GWAS
rs9515212	*COL4A2*	G (0.42)	0.138(0.03)	$8.59E^{-08}$	Systolic and	0.167/0.108	ICBP
					Diastolic blood pressure	0.058/0.375	ICBP
					HDLC	0.005/0.318	GLGC
					LDLC	0.007/0.184	GLGC
					Triglycerides	0.002/0.966	GLGC
					Pericardial adipose tissue volume	NA/**0.011**	VATGen GWAS
					Coronary artery disease	0.01/0.313	CARDIoGRAMplus C4D
					Heart rate	0.003/0.955	Heart rate GWAS
rs1808382	*ACTN4*	T (0.475)	-0.123(0.02)	$1.55E^{-08}$	Systolic and	NA/NA	ICBP
					Diastolic blood pressure	NA/NA	ICBP
					HDLC	NA/NA	GLGC
					LDLC	NA/NA	GLGC
					Triglycerides	NA/NA	GLGC

					Diabetic kidney disease<br>Coronary artery disease	NA/**0.0130**<br>-0.023/**0.0154**	SUMMIT<br>CARDIoGRAMplus C4D
rs3786835	*ACTN4*	A (0.471)	-0.121(0.02)	2.26E^{-08}	Systolic<br>Diastolic blood pressure<br>HDLC<br>LDLC<br>Triglycerides<br>Coronary artery disease<br>Heart Rate<br>Atrial Fibrillation	0.036/0.705<br>0.016/0.8<br>0.016/**0.0213**<br>-0.003/0.615<br>-0.005/**0.064**<br>NA/ **0.022**<br>-0.211/**0.000087**<br>NA/**0.00242**	ICBP<br>ICBP<br>GLGC<br>GLGC<br>GLGC<br>CARDIoGRAMplus C4D<br>Heart rate GWAS<br>2018 AF HRC GWAS
rs16972767	*ACTN4*	A (0.471)	-0.119(0.02)	3.75E^{-08}	Systolic and Diastolic blood pressure.<br>HDLC<br>LDLC<br>Triglycerides<br>Coronary artery disease<br>Heart Rate<br>Atrial Fibrillation	0.03/0.705<br>0.015/ 0.809<br>0.016/**0.0181**<br>-0.003/0.588<br>-0.005/0.07<br>-0.021/**0.0261**<br>-0.211/**0.000074**<br>NA/**0.00242**	ICBP<br>ICBP<br>GLGC<br>GLGC<br>GLGC<br>CARDIoGRAMplus C4D<br>Heart rate GWAS<br>2018 AF HRC GWAS
rs979971	*ACTN4*	T (0.48)	-0.113(0.02)	9.60E^{-08}	Systolic and Diastolic blood pressure<br>HDLC<br>LDLC<br>Triglycerides<br>Coronary artery disease<br>Heart Rate	0.039/0.682<br>0.005/0.933<br>0.016 /**0.0328**<br>-0.004 /0.510<br>-0.005/0.0760<br>NA/**0.026**<br>-0.220/**0.00003**	ICBP<br>ICBP<br>GLGC<br>GLGC<br>GLGC<br>CARDIoGRAMplus C4D<br>Heart rate GWAS

					Atrial Fibrillation<br>Two-hour insulin	NA/**0.003**<br>-0.024/**0.0476**	2018 AF HRC GWAS<br>MAGIC

CARDioGramplus, Coronary ARtery DIsease Genome wide Replication and Meta-analysis (CARDIoGRAM) plus The Coronary Artery Disease (C4D) Genetics; GLGC, Global Lipids Genetics Consortium; MAGIC, Meta-Analyses of Glucose and Insulin-related traits Consortium; SUMMIT, Surrogate markers for Micro- and Macro-vascular hard endpoints for Innovative diabetes Tools; VATgen GWAS, abdominal visceral adipose tissue gen consortium; ICBP; International consortium for blood pressure; NA, not available.

Table XII. Sensitivity analyses using GoDARTS (diabetes).

SNP	Chr.	Position	Gene	A1	A2	GoDARTS		GoDARTS without CAD	
						BETA (SE)	P	BETA(SE)	P
**SNPs associated with arteriolar tortuosity (*TortA*)**									
rs56399312	13	111121981	*COL4A2*	T	C	0.186(0.037)	$6.67E^{-07}$	0.271(0.051)	$3.44E^{-05}$
rs9515212	13	111087563	*COL4A2*	A	G	0.138(0.032)	$1.96E^{-05}$	0.089(0.044)	$4.55E^{-02}$
rs7991229	13	111091995	*COL4A2*	T	G	0.136(0.032)	$2.30E^{-05}$	0.092(0.044)	$3.93E^{-02}$
rs35131825	13	111104752	*COL4A2*	G	A	0.152(0.032)	$3.92E^{-06}$	0.122(0.045)	$7.44E^{-03}$
rs6492273	13	111085494	*COL4A2*	T	C	-0.14(0.032)	$1.40E^{-05}$	-0.094(0.045)	$3.84E^{-02}$
rs9559803	13	111107863	*COL4A2*	A	G	0.150(0.032)	$5.66E^{-06}$	0.125(0.045)	$6.04E^{-03}$
rs79749172	13	111112219	*COL4A2*	T	C	0.155(0.034)	$9.45E^{-06}$	0.143(0.053)	$8.09E^{-03}$
rs9583493	13	111091632	*COL4A2*	C	A	0.137(0.032)	$2.23E^{-05}$	0.092(0.044)	$3.78E^{-02}$
rs9559797	13	111085411	*COL4A2*	G	C	-0.14(0.032)	$2.29E^{-05}$	-0.095(0.045)	$3.52E^{-02}$
**SNPs associated with venular tortuosity (*TortV*)**									
rs1808382	19	39151034	*ACTN4*	G	T	-0.13(0.026)	$2.14E^{-06}$	-0.077(0.037)	$3.53E^{-02}$
rs3786835	19	39155880	*ACTN4*	G	A	-0.13(0.026)	$1.95E^{-06}$	-0.078(0.037)	$3.41E^{-02}$
rs62121814	19	39159729	*ACTN4*	C	T	-0.12(0.026)	$2.98E^{-06}$	-0.075(0.036)	$4.12E^{-02}$
rs16972767	19	39153044	*ACTN4*	G	A	-0.13(0.026)	$3.28E^{-06}$	-0.075(0.037)	$4.11E^{-02}$
rs979971	19	39144244	*ACTN4*	C	T	-0.11(0.025)	$1.30E^{-05}$	-0.071(0.035)	$4.64E^{-02}$

Table XIII. Co-localization of eQTLs with TortV-associated GWAS SNPs in heart and blood vessel tissues using eCAVIAR.

GWAS locus	GWAS SNP	Tissue	Nearest gene	CLPP
Chr19q13.2	rs1808382	Heart Left Ventricle	***CAPN12***	**0.38**
		Heart Atrial Appendage		0.17
		Artery Aorta		0.24
		Artery Tibial		0.13
Chr19q13.2	rs1808382	Heart Left Ventricle	*ACTN4*	0.09
		Heart Atrial Appendage		0.14
		Artery Aorta		0.12
		Artery Tibial		0.06
Chr19q13.2	rs3786835	Heart Left Ventricle	***CAPN12***	0.14
		Heart Atrial Appendage		0.22
		Artery Aorta		0.27
		Artery Tibial		**0.37**
Chr19q13.2	rs3786835	Heart Left Ventricle	*ACTN4*	0.05
		Heart Atrial Appendage		0.09
		Artery Aorta		0.09
		Artery Tibial		0.06

CLPP,co-localization posterior probability ; eCAVIAR, eQTL and GWAS causal variants identification in associated regions ; eQTL, expression quantitative trait loci; *TortV*, retinal venular tortuosity. Higher CLPP in bold text.

Table XIV. Lead SNPs associated with *TortV* are also associated with heart rate in UK Biobank.

SNPs at 19q13.2 *TortV*	Gene	EA	NEA	N	GoDARTS & ORCADES Meta-analysis *TortV*		UK biobank analysis Pulse rate	
					**BETA (SE)**	**P-Value**	**BETA(SE)**	**P-value**
rs1808382	*ACTN4*	T	G	112,008	-0.123(0.021)	$1.55E^{-08}$	-0.208(0.047)	$9.05E^{-06}$
rs3786835		A	G		-0.121(0.022)	$2.26E^{-08}$	-0.209(0.047)	$9.03E^{-06}$
rs16972767		A	G		-0.120(0.022)	$3.75E^{-08}$	-0.210(0.047)	$8.10E^{-06}$
rs979971		T	C		-0.113(0.021)	$9.60E^{-08}$	-0.198(0.047)	$2.79E^{-05}$
**rs11083475**		A	G		-0.108(0.021)	$3.21E^{-07}$	-0.213(0.046)	$4.67E^{-06}$

TortV associated SNPs are strong LD (r^2 >=0.8) with rs11083475 (Bold text), previously reported SNP for elevated resting heart rate.

Table XV. Power calculation for the previously published SNPs associated with CRAE

Study	SNP	MAF	Beta	P-value	N	Power
Jensen *et al.*, 2016	rs1800407	0.05	-0.14	6.5×10^{-12}	28041	84.4%
Sim *et al.*, 2013	rs2194025	0.09	-1.60	2.1×10^{-12}	22661	>85%
	rs3744061	0.44	-0.86	1.7×10^{-10}	22661	>85%
	rs2281827	0.23	-0.70	1.4×10^{-5}	22661	>85%

Major Resources Tables

Animals (in vivo studies) – Not Applicable

Species	Vendor or Source	Background Strain	Sex

Animal breeding – Not Applicable

	Species	Vendor or Source	Background Strain	Other Information
**Parent - Male**				
**Parent - Female**				

Antibodies – Not Applicable

Target antigen	Vendor or Source	Catalog #	Working concentration	Lot # (preferred but not required)

Cultured Cells – Not Applicable

Name	Vendor or Source	Sex (F, M, or unknown)
