# Supplementary figures and images for: Novel Genetic Locus Influencing Retinal Venular Tortuosity Is Also Associated With Risk of Coronary Artery Disease
Source: Arterioscler Thromb Vasc Biol. 2019 Oct 10;39(12):2542–52. doi: 10.1161/ATVBAHA.119.312552 (PMC6882544; doi:10.1161/ATVBAHA.119.312552)

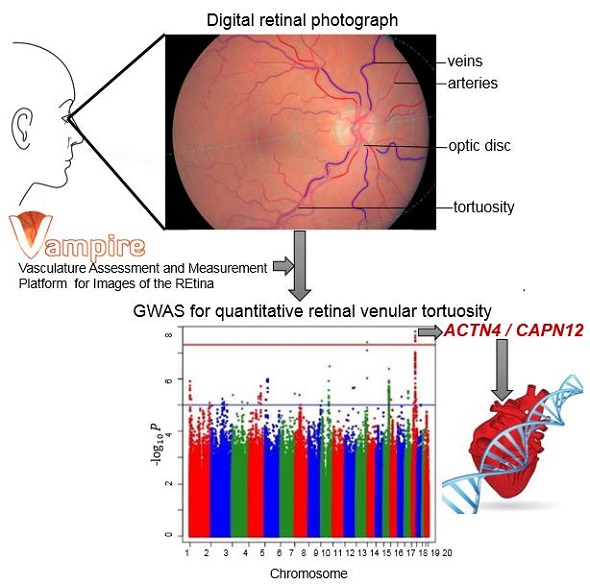

Supplement: Supplementary file 2 [file atv-39-2542-s002.jpg]
